# The effectiveness of behaviour change interventions delivered by non-dental health workers in promoting children’s oral health: A systematic review and meta-analysis

**DOI:** 10.1371/journal.pone.0262118

**Published:** 2022-01-11

**Authors:** Mehreen Riaz Faisal, Masuma Pervin Mishu, Faisal Jahangir, Sabahat Younes, Omara Dogar, Kamran Siddiqi, David J. Torgerson

**Affiliations:** 1 Department of Health Sciences, University of York, York, United Kingdom; 2 Department of Oral Medicine, Margalla Institute of Health Sciences, Rawalpindi, Pakistan; 3 Department of Community Health & Wellbeing, University of Central Lancashire, Preston, United Kingdom; 4 Usher Institute, The University of Edinburgh, Edinburgh, United Kingdom; Griffith University, AUSTRALIA

## Abstract

**Objectives:**

Dental caries is the most common preventable childhood condition. Non-dental professionals and health workers are often well placed to support parents in adopting positive oral health behaviours for their children. The aim of this study was to determine the effectiveness of behaviour change interventions and their individual component behaviour change techniques (BCTs), that were delivered by non-dental professionals and health workers.

**Methods:**

A systematic search of Ovid MEDLINE, PubMed, CINAHL, Cochrane Library, Web of Science, TRoPHI and PROQUEST from inception until March 2021 was conducted. Randomised controlled trials and quasi-experimental studies for improving oral health outcomes in children were included. Quality assessment was carried out using Cochrane Risk of Bias tool and ROBINS-I tool. Publication bias was assessed using funnel plots and Egger’s regression intercept. Effect sizes were estimated as standardised mean difference (SMD) and odds ratio/risk ratio for proportions. Meta-analyses were performed for studies reporting mean decayed, missing, filled surfaces (dmfs) and mean decayed, missing, filled, teeth (dmft) indices. Behaviour change technique coding was performed using behaviour change technique taxonomy v1 (BCTTv1).

**Results:**

Out of the 9,101 records retrieved, 36 studies were included with 28 showing a significant effect either in clinical and/or behavioural/knowledge outcomes. Most studies (n = 21) were of poor methodological quality. The pooled SMD for caries experience showed statistically significant result for caries prevention at surface level -0.15 (95% CI -0.25, -0.04) and at the tooth level -0.24 (95% CI -0.42, -0.07). In 28 effective interventions, 27 individual BCTs were identified and the most frequently used were: *“Instructions on how to perform the behaviour”* and *“Information about health consequences”*.

**Conclusion:**

There is low quality of evidence suggesting non-dental professionals and health workers may help improve oral health outcomes for children. To confirm these findings, further high-quality studies incorporating a variety of BCTs in their interventions for adoption of good oral health behaviours are needed.

## Introduction

Untreated caries of the primary dentition affects 7.8% of the global population with 573 million children affected [1]. Increased severity of the disease is found in those from disadvantaged backgrounds [2] and living in low income countries [3]. Dental caries can have both short and long-term negative impacts on the child in terms of pain and discomfort, difficulty eating and sleeping, which could significantly affect their physical development [4–6] and oral health related quality of Life (OHRQoL) [7, 8]. Furthermore, emergency dental treatment under general anaesthesia can be a source of unnecessary psychological distress and financial burden with consequences extending beyond the family [9, 10]. Dental treatments in UK have been reported to cost NHS £3.4 billion per year [11].

Dental caries is largely preventable and significantly moderated by behaviour [12]. The key behaviours related to dental caries prevention are regular toothbrushing with fluoride toothpaste and limiting sugar consumption [13–16]. The effectiveness of preventive strategies is largely based on a person adopting the healthy behaviour [17]. Children are dependent on their parents or carers for their early childhood developmental needs and so interventions targeting parents for adoption of positive oral heath behaviours for their children can be beneficial in prevention of dental caries [18, 19].

Dental caries initiates early in the life course and with a focus on early prevention, in the recent years, there has been growing emphasis on integrating oral health promotion into primary care and also on task shifting from professional to non-professional workforce [20, 21]. Primary care professionals and health workers such as nurses, midwives and community health workers are often well positioned to deliver oral health advice as they are routinely in contact with expectant mothers and carers of young children [22]. When provided with adequate training and resources, along with the sense of trust and rapport that they share with their community, have proved successful in achieving improved health outcomes for their communities [23].

Many studies have examined the results for integrating oral health promotion of children in existing primary care or health care delivery systems, however, in order to understand how interventions work to produce behaviour change, it is important to identify effective interventions and their components. The behaviour change techniques (BCTs) are identified as those active components of interventions that are designed to change behaviour [24]. This could help in developing new and more effective interventions [25, 26].

Previous reviews have either focussed on type of intervention delivery personnel [22], or interventions targeting mothers of very young children [27]. To date, there has been no attempt at quantifying results to evaluate effectiveness of interventions delivered through non-dental professionals and health workers, and identification of intervention components of effective interventions. Therefore, to address this gap in literature, this review aims to:

1. Determine the effectiveness of behaviour change interventions delivered through non-dental professionals and health workers for young children’s oral health promotion
2. Identify BCTs by coding descriptions of effective interventions using the behaviour change technique taxonomy version 1 (BCTTv1).

## Methods

This review was designed and conducted in accordance with PRISMA guidance (S1 Checklist). The review is registered on PROSPERO (CRD42019139401).

### Eligibility criteria

We included studies, both randomised controlled trials (RCTs) and quasi-experimental studies including pre and post study designs. The following eligibility criteria was used:

- Population: Pregnant women, parents/caregivers of children aged 0–7 years.
- Intervention: Oral health interventions including preventive and behaviour change interventions with either educational component, and/or for improving skills and/or eliciting behaviour change, that were delivered by non-dental nurses or midwives or community health workers or health volunteers or peer support groups.
- Comparison: Any comparator group was considered eligible.
- Outcomes: Any outcome measure that reported clinical oral health status of children indicated by caries index and/or plaque or gingival/periodontal index; and/or change in oral health behaviours such as tooth brushing, dietary habits, use of dental services. Outcomes such as change in oral health related knowledge of parent(s)/caregiver(s) of children, and OHRQoL of parent(s) and/or children were also considered as additional outcomes.

Studies were excluded if they:

- Consisted of interventions focusing on primary outcome of the parent(s)/primary caregiver rather than that of their children.
- Were delivered and/or supervised by teachers at schools.
- Evaluated only the effect of preventive or restorative treatment (e.g. Atraumatic Restorative Technique (ART), fluoride varnish application etc.).
- Were published in languages other than English.

### Data sources and searches

The following databases were searched until March 2021: Both Medline via OvidSP and PubMed (1946- March 2021) to ensure a more comprehensive search [28], Cochrane Library including CENTRAL, CINAHL, Web of Science and TRoPHI, from inception to March 2021. Theses as a source of grey literature were searched via ProQuest. Hand searching of reference lists of previous systematic reviews on similar population was also conducted. Searches were restricted to English language studies only.

The search strategy was developed as combination of key terms and subject headings derived from: *children*, *parents*, *oral health promotion*, *oral health education*, *dental caries*, *toothbrushing*, *primary care*, *nurses*, *community health workers* (S1 File).

### Study selection

Titles and abstracts were reviewed independently by two reviewers (MRF and FJ), against the inclusion and exclusion criteria.

### Data extraction

For each study, data was extracted for: the study title, design, setting, eligibility criteria of participants, sample size, and baseline characteristics of participants and intervention details based on the template for intervention description and replication (TIDieR) checklist [29]. Two reviewers (MRF and MPM) independently reviewed the full text of articles for data extraction purpose with regular meetings to resolve any disagreements.

### Quality assessment

The quality of included studies was assessed by two reviewers independently (MRF and MPM) using the Cochrane risk of bias (RoB) Assessment tool for RCTs in six domains: selection bias, performance bias, detection bias, attrition bias, reporting bias and other bias [30]. The risk of bias in non-randomised studies-of intervention (ROBINS-I) tool was used for quasi-experiments’ assessment on four domains: confounding bias, selection bias, information bias and reporting bias [31].

### Data synthesis

#### Outcomes and effectiveness assessment

Both clinical and behavioural outcome measures were included. A summary statistic, the effect size, was calculated and a narrative synthesis presented. For studies reporting continuous data as outcome measurement, the effect size was presented as Cohen’s d [32]. For studies presenting proportions, odds ratio (OR)/ relative risk (RR) were calculated. For proportions presented in single arm pre-post study designs, relative risk reduction (RRR) was calculated as a measure of the effect size [33].

#### Meta-analysis

Studies included in the review that reported clinical outcomes as measure of caries experience in the form of decayed, filled, missing surfaces (dmfs) and/or decayed, filled, missing teeth (dmft) indices along with variance estimates, were included in the meta-analysis performed.

As there was expectation of considerable heterogeneity due to varying sample sizes and the diversity of outcomes and measures, techniques were used to both examine and limit the impact of heterogeneity: such as test to quantify the heterogeneity, use of random-effects model, sub-group analysis and presentation of prediction intervals (PI).

The I^2^ statistic was used for heterogeneity assessment and a random effects model was used. Sub-group analysis based on intervention type- oral health education only (provided through either verbal, written or audio-visual aids) versus comprehensive intervention (including in addition to oral health education, increased level of support such as either motivational interviewing, provision of toothbrushes/toothpastes, community engagement, and fluoride varnish and sealant application) was performed [34]. Estimated 95% prediction intervals were presented to provide a range in which the point estimate of 95% of future studies will fall, in order to allow for informative inferences to be made from the meta-analyses [35–37]. The outcome was standardised mean difference (SMD) to account for difference in unit of measure for e.g. use of decayed, extracted, filled surfaces (defs) index and not dmfs. Publication bias was assessed using funnel plots and Egger’s regression test [38]. Asymmetric funnel plot or significant Egger’s test were considered an indication for publication bias. All analysis was performed using Stata v17 (StataCorp 2021) using metan package [39, 40].

Due to great variation in how behavioural outcomes were measured and reported, it was not possible to pool them together in a meta-analysis, hence, a narrative synthesis was presented for them.

#### Behaviour change technique coding

Based on the statistically significant effect size, intervention descriptions of effective interventions were coded for BCTs using the BCTTv1 [41]. Where intervention descriptions were not detailed enough to facilitate the coding process, additional materials such as published and unpublished intervention protocol/copies were sought by contacting the authors. Coding was performed by two reviewers [MRF and SY] independently with discussions to resolve disagreement. Both the reviewers undertook training prior to BCT coding (www.bct-taxonomy.com).

Whilst coding the intervention descriptions, where necessary (any further information could not be obtained after two attempts), following coding assumptions were undertaken [42]. When no further details of intervention contents were provided other than ‘health education’ or ‘counselling’, following BCTs were coded: *3*.*1 “social support (unspecified)”* and *5*.*1 “information about health consequences”*. For printed materials (leaflets, pamphlets etc) mentioning provision of ‘information’ or ‘education’ without any further details, they were coded, at the minimum for *5*.*1 “information about health consequences”* and *4*.*1 “instructions on how to perform the behaviour”*.

## Results

**Fig 1:**
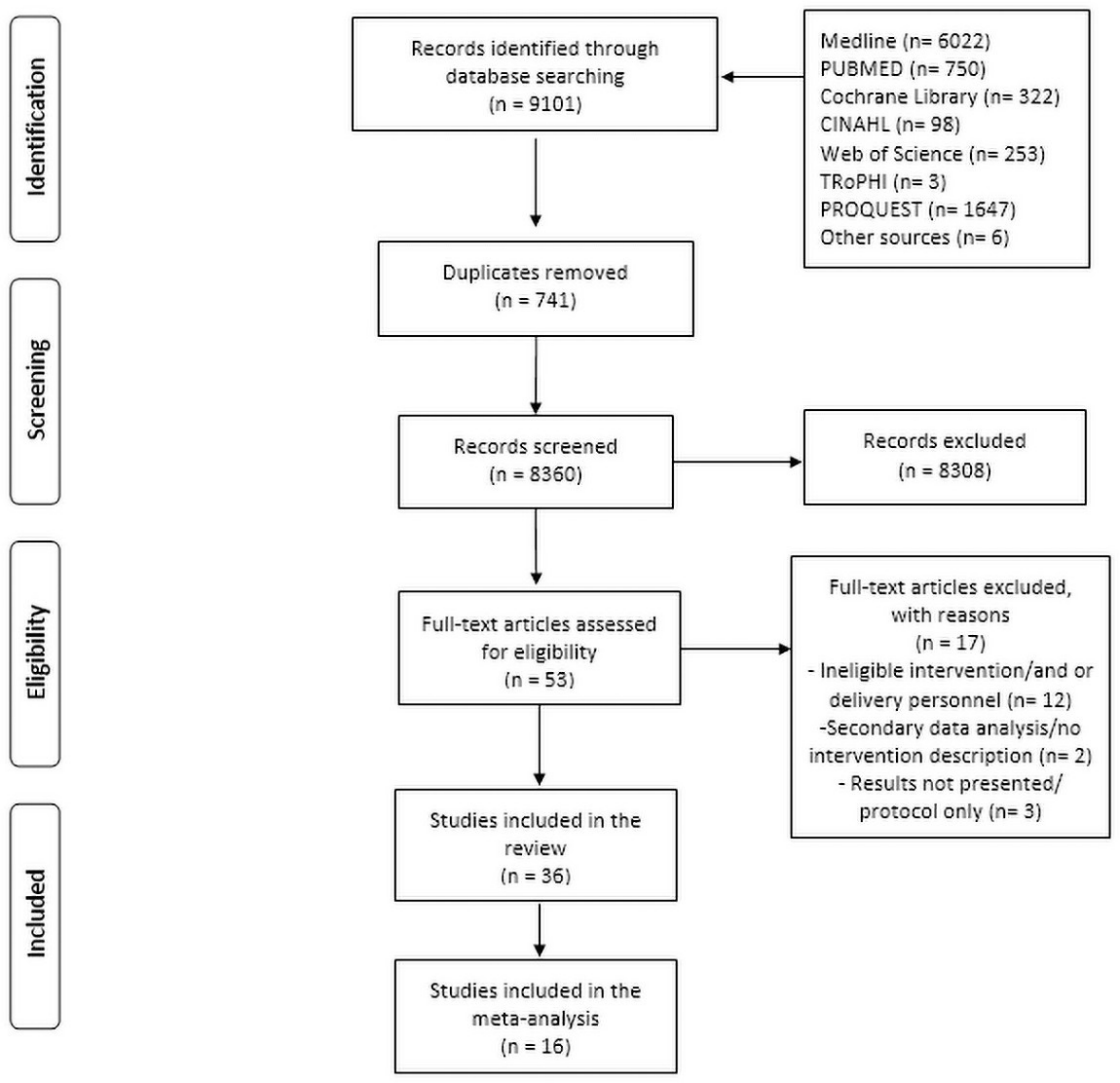
PRISMA flowchart of study selection process.

The search retrieved 9,101 records initially (Fig 1). After removal of duplicates, 8,360 title and abstracts were screened against the eligibility criteria. Fifty-three studies met the inclusion criteria and their full texts were then assessed for inclusion resulting in 36 individual studies that were included in this review (S1 Table).

### Study characteristics

Out of the included studies, 17 were RCTs (47.2%) and 19 were quasi-experimental designs (52.8%) including seven pre-post studies and 12 studies with a comparison or control group.

Studies were conducted in Australia [43–46], Belgium [47], Brazil [48, 49], Cambodia [50], Canada [51, 52], Hong Kong [53], India [54, 55], Iran [56], Ireland [57, 58], Israel [59], Peru [60], Thailand [61, 62], UK [63–66], and USA [67–78]. The mean age of children reported in the included studies ranged from newborns (1–5 days old) to 5.2 years.

### Intervention characteristics

Seven out of 36 studies reported basing their intervention on a theoretical model [43, 47, 56, 61, 62, 69, 72]. In all the reviewed studies, oral health education was a basic component used in combination with other assessments such as caries risk assessment or oral health assessment/screening/ oral examination (n = 6), and other preventive techniques such as fluoride varnish application (n = 6). Many studies reported provision of toothbrushes and toothpastes as part of the intervention (n = 21). Intervention materials pertaining to oral health education consisted of verbal or written material, videos or a combination of these.

The non-dental intervention delivery personnel included combination of physicians (n = 3), nurses (n = 11), midwives (n = 1) dietician (n = 1), health centre staff (n = 3), administration staff (n = 1), vaccination staff (n = 1), health visitors (n = 6), community health workers (n = 7), peers (n = 1) and community members (n = 2).

There was considerable variation in timing, frequency and duration of intervention delivery with most of the studies synchronising with routine visits at the health centre or during child’s vaccination visits (n = 17).

Three studies reported on process evaluation including assessment of intervention fidelity [44, 47, 62], while others reported using quantitative and/or qualitative methods for assessing intervention fidelity.

### Quality assessment

The Risk of Bias assessment for RCTs rated ten as low risk [48, 49, 52, 53, 56, 61, 66, 67, 71, 77], four had unclear risk of bias [43, 64, 65, 69] and two had high risk of bias [70, 74].

In quasi-experimental studies, one study was at low risk of bias [57], seven had moderate risk [45, 50, 51, 63, 73, 72, 75] and rest were classified as having serious risk of bias [44, 46, 47, 54, 55, 58, 59, 62, 68, 76, 78]. The serious risk of bias mostly pertained to measurement bias based on failure to ‘blind’ the outcome assessors and results based on self-reporting of oral health behaviours.

### Intervention effectiveness

Based on the information provided in the published articles effect size estimation was possible for 33 studies (S1 Table).

#### Clinical outcomes

Clinical outcomes were presented using various indices such as dmft or dmfs/defs (n = 20), CAST index (n = 1), or as decay classified as early childhood caries (ECC) (n = 5). Debris index, plaque index, gingival and bleeding indices were also used (n = 4). Out of 21 studies reporting clinical outcomes, 10 studies (47.6%) were found to have significant effect size.

#### Behavioural outcomes

Preventive behaviours such as oral hygiene and dietary behaviours including assessment of sugar consumption, and dental service utilisation were evaluated as part of behavioural outcomes for the purpose of this review. Twenty-four studies (66.7%) reported behavioural outcomes either along with clinical outcomes or as solo outcome measurement. There was great variation in how dietary practices were measured with studies reporting improvement in all or at least some of them [46–48, 51, 53, 58, 59, 62, 64, 65, 72].

Oral health related knowledge and perceptions outcomes were reported in 10 studies and one study reported OHRQoL outcomes.

Effect size calculations were found to be significant for both the two studies that reported behavioural outcomes as mean oral health behaviour scores [69, 76]. For 13 studies that analysed oral hygiene behaviours, nine studies (69.2%) had a significant effect size [46, 53, 55, 58, 59, 61, 62, 65, 72]. A significant effect size was also seen for nine (60%) out of 15 studies that reported dietary behaviours [46–48, 51, 53, 58, 62, 64, 72]. Furthermore, effect size calculations were also found to be significant for seven out of 10 studies (70%) reporting changes in oral health knowledge [44, 46, 54, 62, 71, 76, 72] and four studies out of seven (57.1%) that aimed to improve dental registration or status of being under the care of a dentist [43, 54, 60, 72].

Only one study measured OHRQoL and reported improvement in it [50].

#### Meta-analysis

**Fig 2:**
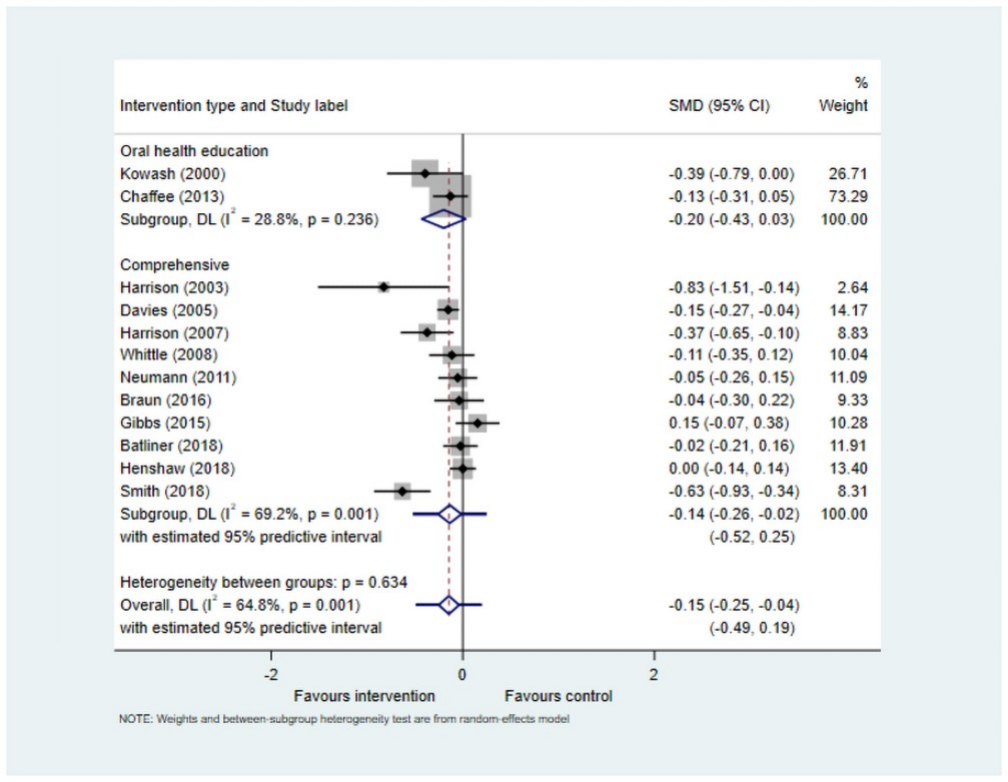
Forest plot for studies reporting caries experience as dmfs.

**Fig 3:**
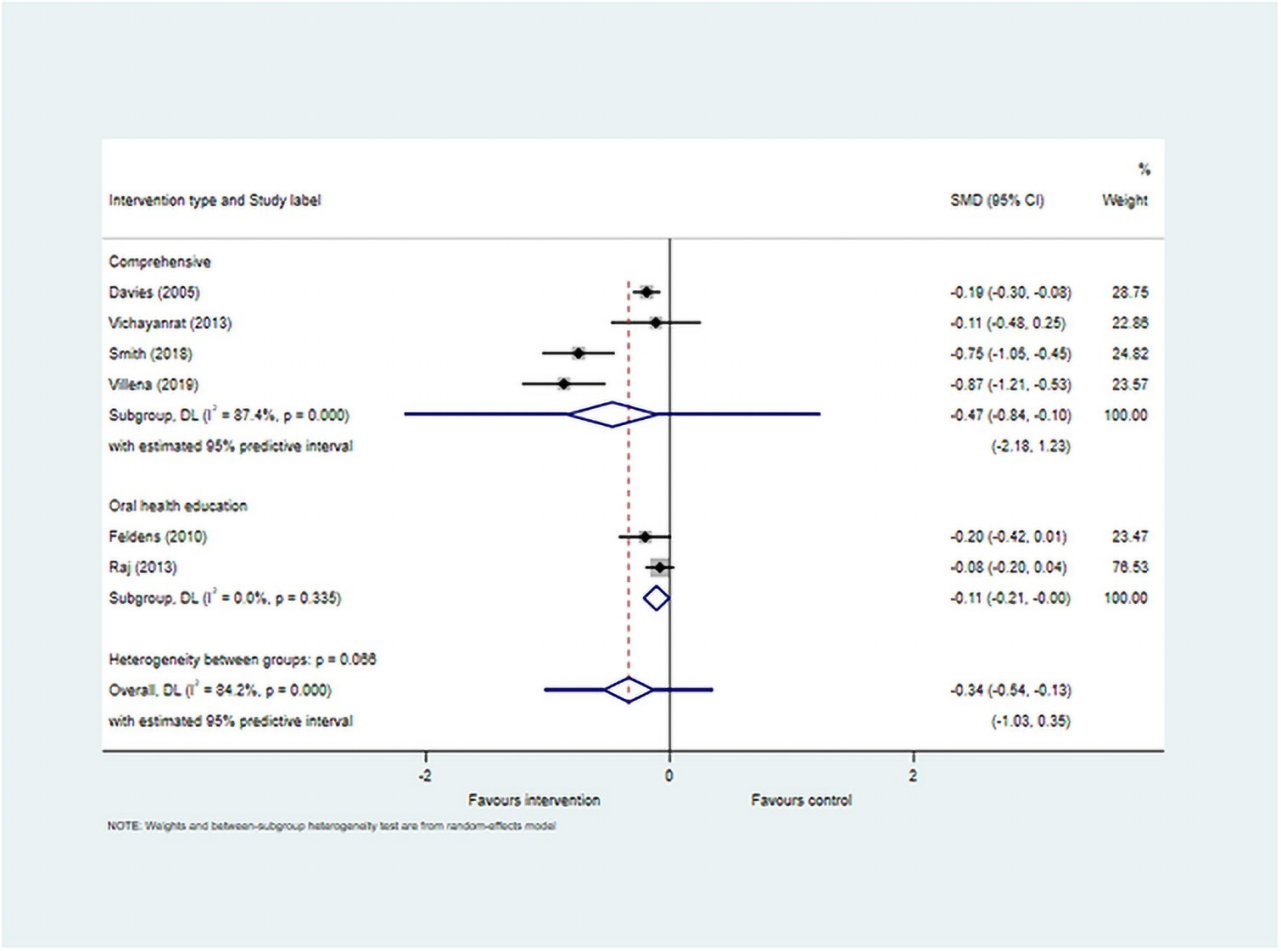
Forest plot for studies reporting caries experience as dmft.

Meta-analyses (Figs 2 and 3) included 12 studies that reported mean dmfs scores, and six studies with mean dmft scores (including two studies that reported both). Use of SMD for meta-analysis resulted in pooled estimate for dmfs -0.15 (95% CI -0.25, -0.04) and for dmft -0.34 (95% CI -0.54, -0.13) both of which were statistically significant.

This suggests there is evidence of effectiveness of interventions in preventing caries when measured at the surface level and the tooth level. However, there was considerable heterogeneity encountered for both the meta-analyses (I^2^ 64.8% and 84.2%respectively), and the 95% prediction interval was not statistically significant for both dmfs (-0.49, 0.19) and dmft (-1.03, 0.35). This can be interpreted to mean that based on the available data, in future studies the SMD could be as low as -0.49 for dmfs and -1.03 for dmft and as high as 0.19 and 0.35 respectively.

The sub-group analysis based on intervention type showed that interventions utilising a more comprehensive approach to promote children’s oral health produced statistically significant reduction in caries experience at both the surface level (SMD -0.14 (95% CI -0.26, -0.02) and at the tooth level (SMD -0.47 (-0.84, -0.10). However, the number of studies in the sub-groups was small and there was high level of heterogeneity observed.

**Fig 4:**
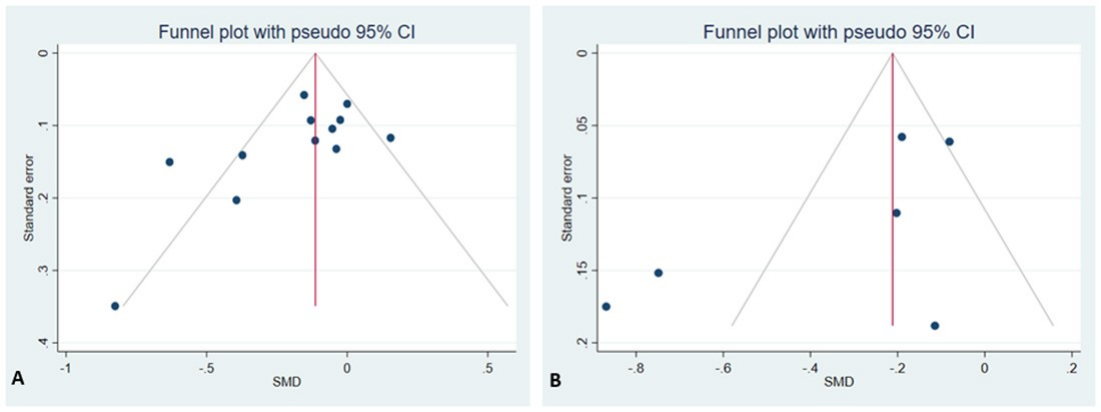
Funnel plots for assessment of publication bias for studies reporting (A) dmfs (B) dmft.

The funnel plot for dmfs showed asymmetry among the studies and also had a significant Egger’s test coefficient (*z* = -2.64, p = 0.008) indicating presence of publication bias. There was no indication of publication bias for dmft reported outcome based on funnel plot symmetry and Egger’s test (*z* = -1.53, p = 0.125) (Fig 4A and 4B).

#### Behaviour change techniques

Twenty-seven distinct BCTs were identified by coding descriptions of effective interventions as indicated by the effect size estimate (Kappa 0.761). All interventions used a combination of BCTs and two most frequently used were– *4*.*1 “Instructions on how to perform the behaviour”* and *5*.*1 “Information about health consequences”*. The most number of BCTs utilised in an intervention was 13 and least was two (Table 1).

**Table 1:** Frequency of BCTs in effective interventions (n = 28).

Behaviour Change Techniques (n = 28)	Frequency in effective interventions	BCT Group based on BCTTv1
**1.1. Goal setting (behaviour)**	4	
**1.2. Problem solving**	7	
**1.4 Action planning**	3	1. Goals and Planning
**1.5 Review behaviour goals**	2	
**1.9 Commitment**	1	
**2.2 Feedback on behaviour**	2	
**2.3 Self-monitoring of behaviour**	1	2. Feedback and Monitoring
**3.1 Social support (unspecified)**	13	
**3.2 Social support (practical)**	8	3. Social support
**4.1 Inst on how to perform the behaviour**	23	4. Shaping knowledge
**5.1 Info about health consequences**	23	
**5.2 Salience of consequences**	1	5. Natural consequences
**5.3 Info about social & environmental consequences**	4	
**6.1 Demonstration of behaviour**	11	6. Comparison of behaviour
**6.3 Info about other’s approval**	1	
**7.1 Prompts & cues**	8	7. Associations
**7.8 Associative learning**	1	
**8.1 Behavioural practice**	4	8. Repetition and substitution
**8.2 Behaviour substitution**	3	
**8.3 Habit formation**	1	
**9.1 Credible source**	2	9. Comparison of outcomes
**9.2 Pros & Cons**	1	
**10.3 Non-specific reward**	2	10. Reward and threat
**10.4 Social reward**	1	
**12.1 Restructuring physical environment**	2	12. Antecedents
**12.5 Adding objects to the environment**	14	
**13.1 Identification of self as role model**	2	13. Identity

## Discussion

This is the first review, we are aware of, to synthesise evidence and identify behaviour change techniques used in interventions for oral health promotion of children through non-dental professionals and health workers. Thirty-six studies were included in the review with the aim of determining which interventions were effective in improving oral health outcomes of children and identifying the BCTs employed by effective interventions.

Although the review identified 28 studies to have produced a significant effect in either clinical and/or behavioural outcomes assessed, only eight were at low risk and seven at moderate risk of bias indicating scarcity of evidence from high quality, low bias studies [22, 27, 79].

As the intervention content differed greatly between studies it was not possible to clearly distinguish the most effective type of intervention. However, it can be positively stated that interventions usually employing a variety of methods such as disseminating oral health education with either provision of dental products, community engagement, dental visits or fluoride varnish application were more effective in producing behaviour change [22, 27, 34].

The observed pooled effect size estimate for prevention of caries at the surface level and the tooth level was small but statistically significant. However, a judgement about its clinical significance would need consideration about other contextual factors including but not limited to existing level of caries in the population and estimation of cost versus the benefits [80].

The medical research council’s (MRC) guidance on developing complex interventions posits health interventions are more likely to be effective if they are based on a theory or a theoretical framework [81]. However, there was poor reporting of theoretical basis of interventions and there is need for more studies to test effectiveness of interventions grounded in theory.

In a systematic review by Cooper et al (2013) which examined effectiveness of primary school-based behavioural interventions for preventing caries in children, similar results with regards to interventions utilising a variety of BCTs were reported [82]. In the current review, a range of three to 13 BCTs per effective intervention was noted. This is an indication of how technically variable the interventions are and only few techniques maybe required in achieving the desired behaviour change for improved oral health outcomes of children. Although this can be a particular advantage in low resource settings [42], it is at the same time, important to consider how they relate to the context for which they are intended. This will ensure the barriers to good oral health behaviours can be addressed adequately.

Traditionally, the focus of oral health promotion has been on transfer of knowledge to change behaviour [83]. In a systematic review assessing health promotion interventions for improvement in oral health of adolescents, the authors reported that more comprehensive interventions that enacted not only at the personal level but also involved wider elements at the family and community level, were more effective in improving oral health outcomes as compared to provision of oral health education alone. These results also support the now widely acknowledged views on shifting the focus from just targeting the individual to a more holistic approach considering the wider familial and societal influences [84]. Thus, interventions targeting a range of barriers can not only support the adoption of good oral health behaviours but also the possibility of their being sustained for a longer time period [83].

Furthermore, although studies reported providing adequate training to the intervention deliverers, it is quite possible that delivery might have been adjusted according to participants’ needs and concerns. For this reason, assessment of intervention fidelity or how well the intervention was delivered as planned, should be an essential part of intervention studies. Testing for intervention fidelity allows for assessment of its mediating role between context and intervention effectiveness and the resultant impact on study outcomes [85]. Failure to evaluate intervention fidelity, also known as Type III error [86] may result in spurious conclusions about intervention effectiveness.

### Strengths

This review systematically appraised and synthesised evidence of oral health promotion interventions delivered through non-dental professionals and health workers and extracted BCTs which were part of effective interventions.

Use of BCTTv1 taxonomy and coding guidelines ensured systematic and consistent coding of intervention descriptions.

### Limitations

Inclusion of only English language publications may have caused relevant studies to be missed during the search process. Furthermore, lack of completeness of intervention descriptions or clarity of reporting along with necessary coding assumptions undertaken, may possibly have caused under or over presentation of some of the BCTs. Also, most of the studies included in the review were at a high risk of bias, and this needs to be considered when interpreting the results.

There was considerable variation between studies, types of interventions and outcomes assessed, leading to high level of heterogeneity encountered in the meta-analyses. Although measures such as use of a random effects model and sub-group analysis were undertaken to explore and address the heterogeneity, it is suggested to exercise caution while interpreting results of the meta-analyses and publication bias.

## Conclusions

Oral health promotion and behaviour change techniques facilitated by primary care workers may help address the global burden of dental caries. The results of this review provide some evidence to support the use of non-dental health professionals and health workers in reducing caries incidence through a variety of BCTs. However, the quality of the existing studies to date is low with a high risk of bias. Future research and robust clinical trials using standardised taxonomy may improve the generalisability of these findings.

## Supporting information

S1 Checklist PRISMA checklist. (DOC)

S1 File Search strategy. (DOCX)

S1 Table Summary table. (DOCX)
