# Supplementary material for: The effectiveness of behaviour change interventions delivered by non-dental health workers in promoting children’s oral health: A systematic review and meta-analysis
Source: PLoS One. 2022 Jan 11;17(1):e0262118. doi: 10.1371/journal.pone.0262118 (PMC8751985; doi:10.1371/journal.pone.0262118)
Supplement: S1 File — (DOCX) [file pone.0262118.s002.docx]

**Medline search strategy**

Set Search Statement

1 exp child/ or exp child, preschool/ or exp infant/ or exp parents/

2 child*mp.

3 child*.mp.

4 infan*.mp.

5 toddler.mp.

6 mother*.mp.

7 mother*.mp.

8 pregnan*.mp.

9 pregnan*.mp.

10 parent*.mp.

11 dyad.mp.

12 famil*.mp.

13 newborn.mp.

14 early childhood.mp.

15 or/1-14

16 oral health education.ti,ab.

17 oral health education.tw.

18 oral health education.mp.

19 oral health promotion.mp.

20 exp Health Promotion/ or exp Dental Care/ or exp Oral Health/ or exp Health Education, Dental/ or exp Dental Caries/

21 community intervention.mp.

22 community outreach.mp.

23 exp Primary Health Care/

24 Preventive Dentistry/

25 oral health advi?e.mp.

26 (health adj2 (promot* or advi?e or educat* or practic* or improv*)).mp.

27 or/16-26

28 dental decay.mp.

29 exp Dental Caries/

30 caries.mp.

31 exp Dental Plaque/

32 toothbrush*.mp.

33 exp Oral Health/ or exp Periodontal Diseases/ or exp Dental Care/ or exp Dental Plaque/ or exp Gingivitis/ or exp Dental Caries/ or exp Oral Hygiene/ or exp Toothbrushing/

34 (oral health adj knowledge).mp. [mp=title, abstract, original title, name of substance word, subject heading word, floating sub-heading word, keyword heading word, organism supplementary concept word, protocol supplementary concept word, rare disease supplementary concept word, unique identifier, synonyms]

35 (oral health adj knowledge).mp.

36 exp Oral Hygiene/

37 oral care.mp.

38 dental health.mp.

39 oral health.mp.

40 exp Oral Health/

41 (oral health adj2 behavio*).mp. [mp=title, abstract, original title, name of substance word, subject heading word, floating sub-heading word, keyword heading word, organism supplementary concept word, protocol supplementary concept word, rare disease supplementary concept word, unique identifier, synonyms]

42 (oral health adj2 behavio*).mp.

43 exp Oral Hygiene/ or exp Dental Plaque/ or exp Toothbrushing/ or exp Gingivitis/ or exp Dental Devices, Home Care/

44 Oral health related quality of life.mp.

45 Oral health related quality of life.ti,ab.

46 OHRQoL.mp.

47 or/28-46

48 health visit*.mp.

49 health visit*.ti,ab.

50 community health worker*.mp.

51 exp Community Health Services/ or exp Community Health Workers/

52 exp Health Personnel/

53 exp Midwifery/ or lady health visitor*.mp.

54 exp Community Health Nursing/ or community health visitor*.mp.

55 health aide.mp.

56 exp NURSE PRACTITIONERS/ or exp NURSE MIDWIVES/

57 exp Midwifery/

58 exp Nurses' Aides/ or exp Allied Health Personnel/

59 exp Nurses' Aides/ or support worker.mp. or exp Allied Health Personnel/

60 home visit.mp.

61 nurse*.mp.

62 Education, Nursing/ or Nursing/ or Public Health Nursing/ or Neonatal Nursing/ or Pediatric Nursing/ or Maternal-Child Nursing/ or Community Health Nursing/ or Primary Care Nursing/ or Obstetric Nursing/ or Nursing Staff/

63 ((nurs* adj2 educat*) or (midwi* adj2 educat*) or nurs*adj2 train or (midwi* adj2 train*) or (nurs* adj2 practic*) or (midwi* adj2 practic*)).mp.

64 home visit*.mp.

65 lay health worker*.mp.

66 or/48-65

67 randomized controlled trial.pt.

68 randomized.ab.

69 randomly.ab.

70 trial.ab.

71 groups.ab.

72 exp Feasibility Studies/

73 feasibility study.ti,ab.

74 pilot study.ti,ab.

75 exp Pilot Projects/

76 or/67-75

77 exp animals/ not humans.sh.

78 76 not 77

79 pretest-posttest study.mp. [mp=title, abstract, original title, name of substance word, subject heading word, floating sub-heading word, keyword heading word, organism supplementary concept word, protocol supplementary concept word, rare disease supplementary concept word, unique identifier, synonyms]

80 pretesting.mp. [mp=title, abstract, original title, name of substance word, subject heading word, floating sub-heading word, keyword heading word, organism supplementary concept word, protocol supplementary concept word, rare disease supplementary concept word, unique identifier, synonyms]

81 pre-post tests.mp. [mp=title, abstract, original title, name of substance word, subject heading word, floating sub-heading word, keyword heading word, organism supplementary concept word, protocol supplementary concept word, rare disease supplementary concept word, unique identifier, synonyms]

82 quasi experimental design.mp. [mp=title, abstract, original title, name of substance word, subject heading word, floating sub-heading word, keyword heading word, organism supplementary concept word, protocol supplementary concept word, rare disease supplementary concept word, unique identifier, synonyms]

83 quasi experimental study.mp. [mp=title, abstract, original title, name of substance word, subject heading word, floating sub-heading word, keyword heading word, organism supplementary concept word, protocol supplementary concept word, rare disease supplementary concept word, unique identifier, synonyms]

84 quasi experimental study design.mp. [mp=title, abstract, original title, name of substance word, subject heading word, floating sub-heading word, keyword heading word, organism supplementary concept word, protocol supplementary concept word, rare disease supplementary concept word, unique identifier, synonyms]

85 repeated measurement.mp. [mp=title, abstract, original title, name of substance word, subject heading word, floating sub-heading word, keyword heading word, organism supplementary concept word, protocol supplementary concept word, rare disease supplementary concept word, unique identifier, synonyms]

86 repeated measurements.mp. [mp=title, abstract, original title, name of substance word, subject heading word, floating sub-heading word, keyword heading word, organism supplementary concept word, protocol supplementary concept word, rare disease supplementary concept word, unique identifier, synonyms]

87 repeated measures.mp. [mp=title, abstract, original title, name of substance word, subject heading word, floating sub-heading word, keyword heading word, organism supplementary concept word, protocol supplementary concept word, rare disease supplementary concept word, unique identifier, synonyms]

88 time series.mp. [mp=title, abstract, original title, name of substance word, subject heading word, floating sub-heading word, keyword heading word, organism supplementary concept word, protocol supplementary concept word, rare disease supplementary concept word, unique identifier, synonyms]

89 79 or 80 or 81 or 82 or 83 or 84 or 85 or 86 or 87 or 88

90 78 or 89

91 15 and 27 and 47 and 66 and 90
