# Supplementary material for: The effectiveness of behaviour change interventions delivered by non-dental health workers in promoting children’s oral health: A systematic review and meta-analysis
Source: PLoS One. 2022 Jan 11;17(1):e0262118. doi: 10.1371/journal.pone.0262118 (PMC8751985; doi:10.1371/journal.pone.0262118)
Supplement: S1 Table — (DOCX) [file pone.0262118.s003.docx]

**S1 Table. Summary Table of Included Studies**

| **Author**  **&**  **Study design** | **Study location & aims** | **Participant**  **characteristics** | **Intervention delivery personnel** | **Intervention** | **Outcome measures** | **Findings** | **Quality** |  |
| --- | --- | --- | --- | --- | --- | --- | --- | --- |
| **Randomised Controlled Trials** | | | | | | | | |
| **Kowash et al. (2000)**  **RCT (4 arms + 1 control)** | **United Kingdom**  To determine the effect of dental health education (DHE) on caries incidence in infants, through regular home visits by  trained DH Educators over a period of 3 years. | ***Intervention:***  ***Baseline (n= 228 dyads)***  Group A: 60  Group B: 59  Group C: 60  Group D: 49  ***Followup***  Group A: 45  Group B: 47  Group C: 51  Group D: 36  ***Control:***  n=55 dyads  ***Children***  Intervention group A-D:  Mean age (SD): 11.4 (3.4) months at baseline  Comparison group E  Approached only at 3 year follow up  ***Mothers***  Mean age (SD): 29 (5.3) years | 2 Dental Health Educators specially trained every year. One was a Dental Therapist and the other a Paediatric Nurse | ***Intervention***  Oral health education  Diet and Oral hygiene (OH) advice given using structured interview and counselling for 15 mins. The main message was to substitute bottle with feeder cup;  brush child’s teeth twice a day with fluoride toothpaste and visit a  dentist regularly. Each mother, on every visit, was given the opportunity to ask questions and further advice given  accordingly  ***Intervention group***  Group A: DHE on diet + briefly OH  Group B: DHE on OH + briefly on diet  Group C: DHE equally on diet and OH  Above groups received intervention every 3 months for 1st two years. and then twice a year in the third year  Group D: DHE on diet and OH once a year for 3 years.  ***Comparison group***  Did not receive anything. Those initially eligible but not selected were traced and if living in the locality were examined at 3 year follow up to act as controls. | ***Clinical***  Caries status, periodontal status  Measured at 3 yrear follow up  ***Behavioural***  Frequency of drinking and sweet consumption  Frequency of toothbrushing and dental visits | ***Clinical***  There was significant difference reported in mean dmfs for Group A vs. Group E (0.29 (1.64) vs 1.75 (5.09) (p <0.001)  Effect size (d): -0.37 (-0.77, 0.03) not significant.  Only (3%) in the study groups had gingivitis (all in diet group A)  Effect size Group A vs. Group E:  OR 0.27 (0.07, 1.05) not significant  Poor oral hygiene (debris +calculus) (three in group A and six in group D)  Effect size Group A vs Group E:  OR 0.08 (0.02, 0.28)  Group D vs Group E:  OR 0.31 (0.13, 0.77).  ***Behavioural***  Significant differences between each intervention group with Group E for all reported behaviours (p <0.001).  Effect size (OR): not significant for any behaviours for intervention groups vs control group E except for frequency of toothbrushing (more than once/day) for which OR was highly significant (<0.0001) for each intervention group vs. group E. | Unclear risk |  |
| **Davies et al. (2005)**  **Clustered RCT** | **United Kingdom**  To assess the effects of a multi-stage dental health promotion programme in reducing Early Childhood Caries (ECC) | Baseline, n=839 in intervention group and n= 706 in control group  Total 1545 children examined at follow up  ***Intervention:*** Out of the total 649 children had attended the clinic at intervention site  Mean age 3.97  Males: n=432 (51.5%)  Females: n= 405 (48.3%)  ***Control:*** Out of the total 558 children attended clinic at control site.  Mean age: 4.02 years  Males: n= 359 (50.8%)  Females: n= 344 (48.7%) | Health Visitor or Practice Nurse | ***Intervention group***  Oral health education + OHP Kit  5 stages of intervention:  1. At 8 months visit to health centre a gift bag containing trainer cup, leaflet was given and oral health advice by HV.  2. At 12-15 months visit parents were given by HV or practice nurses gift bag containing 1450 ppm fluoride TP and TB.  3. at 18 months children were invited by post to attend local dental clinic and where they were provided Fluoride TP and TB. If not attended within 2 months then it was posted to them  4, 5. Fluoride TP and TB + leaflet posted to them when they were 26 and 32 months old.  ***Control group***  Received usual care | ***Clinical***  Oral examinations at 3-4 years of age for mean dmft, mean dmfs and proportion of children with caries and nursing caries  ***Behavioural***  Questionnaire at 21 months' age to asses:  Use of bottle, trainer cups, sugared drinks  Age of initiation of toothbrushing and frequency of toothbrushing | ***Clinical***  Mean dmft of intervention children vs control was significantly lower (1.17 vs 1.72, p= 0.001)  Effect size (d): -0.19 (-0.3, -0.08).  Mean dmfs of intervention vs control was significantly lower (2.58 vs 3.75, p= 0.008)  Effect size (d): -0.15 (-0.27, -0.04).  ***Behavioural***  Significant differences reported between intervention and control group for ceased Bottle use, only sugared drinks given in cup when used, toothbrushing started before 1^st^ birthday and brushing twice daily (p= 0.04, 0.02, 0.02 and <0.01 respectively).  Effect size (OR) was found to be significant only for use of sugared drinks given in cup when used (OR 0.35 (0.16, 0.77) p= 0.0089. | Unclear risk |  |
| **Vachirarojpisan et al. (2005)**  **Clustered RCT** | **Thailand**  To evaluate process and outcome of a participatory Dental Health Education programme for preventing ECC | ***Intervention***:  Baseline n= 11 health centres, 270 children  Mean age (SD): 12.09 (3.66) months  Males: n= 120 (56.3%)  Females: n= 93 (43.7%)  Mother's n= 183, age (SD): 27.64 (6.42) years  Follow up n= 213 children  ***Control:***  n= 10 health centres, 250 children.  Mean age (SD): 12.24 (3.78) months  Males: n= 96 (50.3%)  Females: n= 95 (49.7%)  Mother's n= 166, age (SD): 27.49 (6.39) years  Follow up n= 191 children | 17 staff members at 11 health centres | ***Intervention group***  Oral health discussion + TB and TP  Group discussions by trained health centre staff with a group of  6-8 parents/caregivers on their children’s oral health and causes and prevention of ECC, based on empowerment model of Health Education and distribution of free toothbrushes toothpastes of 500 ppm. Provided at 3 times at 3 months’ interval.  ***Control group***  Routine DHE programme  based on didactic teaching about ECC prevention and distribution of free toothbrushes Coinciding with vaccination programme at age of 9 months and 18 months. | ***Clinical***  Caries increment reported as mean cavitated, mean non-cavitated and combination of both as ECC  ***Behavioural***  Habit of falling asleep with bottle, night time feeding and sweet food consumption between meals of drinking and sweet consumption  Performance of any toothbrushing, parents’ brushing their children’s teeth, use of fluoride TP and use of proper amount of TP | ***Clinical***  No significant differences reported for mean cavitated, non-cavitated and ECC between intervention and control groups (p>0.05).  ***Behavioural***  Statistically significant improvement for all oral hygiene behaviours assessed (p<0.001). Effect size (OR) significant as well.  The dietary behaviours were not found to be significantly different between intervention and control groups (p >0.05). Effect size (OR) non-significant as well. | Low risk |  |
| **Harrison et al. (2007)**  **Randomised Controlled Trial** | **Canada**  To test motivational interviewing (MI) to prevent early childhood caries; and to use  Poisson regression for data analysis | ***Intervention:***  Baseline n=122 dyads  Follow up n= 105 dyads  ***Control:***  Baseline n= 118 dyads  Follow up n= 100 dyads  ***Children:***  Intervention: mean age (SD) 10.8 (5.3) months  Boys: n= 122 (57%)  Control: mean age (SD) 12.1 (5.3) months  Boys: n= 118 (52%) | 3 local South Asian women trained as ‘MI counsellors’ | ***Intervention group***  OHE- MI counselling + pamphlet and video  One 45-minute counselling session, 2 brief follow-up telephone calls at 2 weeks and 1 month after initial contact. Then four follow-up telephone calls up to 6 months after the initial contact and 2 postcard reminders.  A pamphlet on infant oral health  designed by the local health unit dental  staff but modified to include strategies  to prevent ECC appropriate to the South  Asian community. Mothers also watched  an 11-minute educational video, “Preventing Tooth Decay for Infants and Toddlers.” Both pamphlet and video, available in English and Punjabi, also recommended that parents take their child to PICS for fluoride varnish applications.  ***Control group:***  The pamphlet + video same as in intervention and recommendation to take their children for fluoride varnish application to PICS. | ***Clinical***  Oral examination done at 2 years follow up reporting mean dmfs and mean dmfs+white spots. | ***Clinical***  Statistically significant difference of mean dmfs between intervention and control group (3.35 (7.8) vs 7.59 (14.2) p= 0.001  Effect size (d)= -0.37(-0.65, -0.09) and for dmfs + white spots (3.52 (8) vs. 7.91 (14.2) p= 0.01  Effect size (d)= -0.38 (-0.66, -0.11). | Low risk |  |
| **Whittle et al. (2008)**  **Randomised Controlled Trial** | **United Kingdom**  To determine the effect of oral health education carried out by a specially trained health visitor on the dental health of young children | ***Intervention:***  250 children  3 year follow up n= 181  5 years follow up n= 147  Mean age: 5.13 years  ***Control:***  251 children  3 year follow up n= 171  5 year follow up n= 129  Mean age: 5.20 years | Health visitors | ***Intervention-***  Home visits with dental advice + kit  First visit comprised of dental advice and leaflet along with toothbrush and tooth paste being given. In the 2nd visit when child was 20 months old discussions focused on child's diet and toothbrushing, giving of the leaflet and toothpaste and toothbrush.  The dental advice reinforced through leaflets was based on reduction of sugars and increased consumption of fruits and vegetables, initiation of toothbrushing and advice on toothbrush and toothpaste.  ***Control***  Routine home visits  Usual dental advice relating to getting child registered, toothbrushing and avoiding sugary drinks in face to face. No printed material provided | ***Clinical***  dmfs at 3 years and 5 years of age of children | ***3 year's age follow up***  No statistically significant results reported for difference in mean ds, ms and fs in intervention vs control groups.  ***5 years' age follow up***  A census group was also used for comparison.  Statistically significant differences were reported in the study for INT vs. census group for mean ds, ms and mean dmfs (3.35 vs. 4.71, 0.37 vs. 0.87 and 3.99 vs. 5.94 respectively).  However, the effect size (d) calculation for INT vs. census at 5 years of age was not statistically significant.  ds: -0.17 (-0.33, 00)  ms: -0.14 (-0.31, 0.03)  dmfs: -0.21 (-0.50, 0.08) | Low risk |  |
| **Mohebbi et al. (2009)**  **Cluster RCT** | **Iran**  The study evaluated the impact of the 6-month educational  intervention aimed at preventing dentinal and enamel  caries in 12- to 15-month-olds. | ***Health centres:***  n= 18 (6 in each group)  ***Group A:*** n= 77 dyads at baseline and n= 55 at follow up  ***Group B:*** n= 85 dyads at baseline and n= 59 at follow up  ***Control:*** n=80 dyads at baseline and 63 at follow up  ***Children***  Mean age 12.3 months with 50% males. | Vaccination Staff (n= 36) | ***Intervention groups***  Oral health education  Pamphlet in local Persian language written in plain simple language illustrated with baby pictures and use of 'happy colours'.  **Group A** received pamphlet with 5 minutes verbal instructions + 2 phone call reminders 2 months apart + instructions to be on time in order to reduce drop outs.  **Group B** received only the pamphlet without any other verbal instructions or phone reminders.  ***Control group***  After the trial, mothers received the same pamphlet on caries prevention from the vaccination health staff. | ***Clinical***  Difference in caries increments at baseline and 6months follow-up by assessment of number of dt and de and percentages of children developing new dt and de.  ***Behavioural***  Brief interviews conducted to explore mother’s perception of intervention effectiveness for changing behaviours. | ***Clinical***  No significant difference in dt for Group A vs. C (0.1 (0.6) vs. 0.2 (0.7), p= 0.188 and Group B vs. C (0.1 (0.1) vs. (0.2 (0.7), p= 0.265)  Effect size (d): A vs. C -0.29 (-0.65, 0.08), B vs. C -0.20 (-0.55, 0.16).  Significant difference for de between group A 0(0) vs Control (0.4 (0.7), p <0.001  Effect size (d): -0.78 (-1.15, -0.40)  Group B was 0.2 (0.6) vs. 0.4 (0.7) in controls, p=*0.06*.  Effect size d: -0.31 (-0.66, 0.05)  Logistic Regression analysis controlling for background factors:  OR for developing new de or dt was 0.1 (95% CI 0.0–0.4) for group A and 0.4 (95% CI 0.2–1.0) for group B. | Low risk |  |
| **Feldens et al. (2010)**  **Randomised controlled trial** | **Brazil**  To investigate the effectiveness of home visits advising  mothers about healthy feeding practices during the first year of life on the occurrence of early childhood caries and severe early childhood caries at 4 years  of age. | ***Intervention:*** n=200 dyads at baseline and 157 at follow up  Mean age (SD): 50.6 (1.7) months  Males: n= 85 (60.3%)  ***Control:*** n=300 dyads at baseline and 219 at follow up.  Mean age (SD): 50.4 (1.7) months  Males: n= 110 (55.3%)  ***Mothers***  Mean age at child’s birth 25.7y | 12 Field workers | ***Intervention group***  Oral health education  Verbal dietary, healthy breast-feeding and weaning advice (based on WHO recommendations) was given to mothers within 10 days of child's birth and monthly upto 6 months then 8,10,12 months, in informal manner considering mother's opinions and concerns. Instructions on preparation and recipes of  complementary and healthy food were provided.  ***Control group***  Received routine assistance by paediatrician and dietary advice related to oral health upon completion of research period. | ***Clinical***  Proportion of children with ECC (presence of any decay)  Secondary outcomes: Occurrence of severe early childhood caries (S-ECC)  The number of affected  teeth (d1+mft).  Assessment carried out within 1month after the 12month home visit.  ***Behavioural***  Dietary behaviour (onset, duration and frequency of feeding practices) at 6 and 12months collected during interviews by nutrition students. | ***Clinical***  The proportion of children with ECC (main outcome) was 53.9% (76 ⁄ 141) across the intervention group and 69.3% (138 ⁄ 199) among controls  Effect size: RR 0.78 (0.65–0.93) for children ECC)  S-ECC was present in 41 (29.1%) intervention group children and 85 (42.7%) controls.  RR (0.68 (0.50–0.92) for SECC (reported in study.  Mean number of affected teeth (dmft) was found to be significantly different between intervention vs. control group (3.25 vs. 4.15, p= 0.023)  Effect size (d): -0.20 (-0.42, 0.01).  ***Behavioural***  Significant differences reported between proportion of children in intervention and control groups for ‘age of sugar introduction (>6 months or more)’ (p= 0.010), ‘no. of daily meals or snacks at 1 year (>8)’ (p= 0.035) and ‘consumption of high density of sugar in foods at 1 yr’ (p= 0.002).  ES (OR): 2.37 (1.26. 4.44), 0.51 (0.30, 0.85) and 2.4 (1.41, 4.08) respectively.  No significant difference was reported for ‘night time bottle use at 1 year’ p= 0.382)  ES (OR): 0.80 (0.51, 1.24). | Low risk |  |
| **Neumann et al. (2011)**  **Cluster Randomised controlled Trial** | **Australia**  To evaluate the effectiveness of a community-based intervention  to improve the oral health of children in non-fluoridated rural Victoria, Australia. | ***Intervention***  n= 482 at baseline and n= 146 at 3 years' followup  Females (baseline) n= 224 (46%)  ***Control***  n= 433 at baseline and 246 at 3 years' follow up.  Females (baseline) n=207 (48%). | Maternal and child health nurses (MCHN) | ***Intervention group:***  Oral health promotion  An oral health starter kit (OHSK), which included an age-appropriate toothbrush,  toothpaste and educational information for parents outlining key evidence-based oral health promotion messages. These kits were distributed to all families with infants/toddlers in the intervention LGAs (regardless of whether they agreed to participate in the study or not). In addition, an oral health promotion training programme was provided for the MCHNs in these LGAs along with posters, pamphlets and a video/DVD where required.  ***Control group:***  Usual care | ***Clinical***  Proportion of children with  caries reported through number of decayed, extracted or filled surfaces (defs), and the Significant Caries Index (SiC^10^ and SiC^30^), which is the mean defs in the 10% and 30% of the children with the highest caries experience, all calculated both including and excluding the pre-cavitated surfaces at each of the subsequent annual exams  (exam 1, 2 and 3) | ***Clinical***  When adjusted for age (children in intervention group were slightly younger than those in the control group), there was no statistically significant difference between the intervention vs. control groups for mean defs, SiC^30^ qnd SiC^10^ index. | Unclear risk |  |
| **Chaffee et al. (2013)**  **Cluster randomised controlled trial** | **Brazil**  To estimate the caries impact of providing training in infant feeding guidelines to workers at Brazilian public primary care clinics. | ***Intervention***  9 clinics, 360 children enrolled and 237 children in follow up.  Mean age (SD) 3.2 (0.2) years  Males: n= 119 (50.2%)  Mother's mean age at delivery: 27.1 (6.7) years  ***Control:***  11 clinics, 355 children at baseline and 221 children  in follow up.  Mean age (SD) 3.2 (0.2) years  Males: n= 114 (51.6%)  Mother's mean age at delivery: 25.7 (6.6) years | Healthcare professionals including physician, nurses  and administrative staff | ***Intervention group***  Oral health education  Dietary counselling for pregnant/lactating women aimed to improve oral health outcomes of their children. Pamphlets were also distributed.  ***Control group***  Usual practice of maternal counselling at practitioner’s discretion. | ***Clinical***  dmfs, and Severe ECC (S-ECC) at 2-3 years of age.  ***Behavioural***  Dental visiting at 2-3 years of age of child. | ***Clinical***   - The Relative Risk (RR) for ECC  \| 0.92 (0.75, 1.12); S-ECC 0.87 (0.64, 1.19) \| \| --- \|   and cavitated decay 0.88 (0.66, 1.17) was not statistically significant.   - No statistically significant difference in mean dmfs (any decay) between intervention vs. control groups (2.8 (5.4) vs. 3.6 (6.9), p=0.25).   Effect size (d): -0.13 (-0.13, 0.05).  ***•*** No statistically significant difference in mean dmfs (cavitated decay only) between intervention vs. control groups (2.1 (5.0) vs. 3.0 (6.8), p= 0.18).  Effect size (d): -0.15 (-0.33, 0.03).    ***Behavioural***   - 26.6% reported previously visiting a dentist, and this was not significantly different between the 2 groups. | Low risk |  |
| **Mattheus et al. (2014)**  **Randomised controlled trial** | **USA**  To investigate the effects of oral health promotion provided by primary care providers on parental oral health beliefs and behaviours. | ***Intervention***:  n= 44  ***Control***:  n= 40  Parents of children aged 6-15 months | Paediatric Nurses | Both the groups received standard care including caries risk assessment, oral health examination, fluoride varnish application, anticipatory guidance, attempt to refer to a dental home and a toothbrush provided to the child.  ***Intervention group***  In addition, received enhanced oral care including: extensive oral health history and caries risk assessment, medical history, and an oral health examination, Detailed child and family oral health education was discussed Detailed child and family oral health education was discussed with a handout focusing on common ECC risk factors. A tooth brush was provided during the child’s first enhanced oral health visits, along with education on how to brush, proper use of fluoride toothpaste and the importance of regular brushing. Additionally, a sippy cup was provided at the second visit to reinforce proper oral health beliefs and behaviors, which included information of foods to avoid for the prevention of caries development. At each visit the family was given dental provider information with an attempt to refer them for future assessment and care. | ***Behaviour and Knowledge***  Parental beliefs  about oral health and  parental oral health behaviours for their children 6 to 15 months of age assessed at 6 months follow up | Only p values for difference in scores provided for both the groups for which reason it was not possible to perform the effect size calculations. | High risk |  |
| **Hallas et al. (2015)**  **Randomised controlled trial** | **USA**  To determine the oral health hygiene knowledge of mothers of newborns and the effectiveness of an oral health education program on the oral health of infants at 6 and 12months | ***Intervention:***  47 dyads  ***Comparison:***  47 dyads  ***Children***  Infants between 1-5 days old when mother-child enrolled in the study | Not explicitly mentioned but possibly nurses/midwives | ***Intervention group***  Oral health education + goody bag  Mothers watched 8-minute DVD in postpartum rooms and after that they received the goody bag containing Oral hygiene kit for newborn and oral health brochure  ***Control group***  Standardized 8-minute DVD on nutrition for newborns and infants and oral hygiene kit for newborn and oral health brochure. | ***Clinical***  Oral health assessment and Caries Risk Assessment Tool (CAT) used to assess overall risk of caries  At 6- and 12-months post-partum.  ***Behavioural***  Questionnaire at baseline and 6 months follow-up to assess mother’s oral health knowledge, beliefs and practices.  . | ***Due to high level of attrition, assessments could not be carried out fully as planned.***  ***Clinical***   - Results of oral health assessment at 6 and 12 months showed infants (n=10) to be cavity free although they were at high risk of ECC as assessed by Caries Risk Assessment Tool (CAT).   ***Behavioural***   - Baseline results indicated mother’s lack of oral health knowledge about infants oral health care. | High risk |  |
| **Leung et al. (2015)**  **Cluster randomised controlled trial** | **Hong Kong**  To reported the effectiveness The Healthy Start Home Visit Program based on and expanded from HOPE (Leung et al., 2011) to include physical development issues, in addition to  learning and psychosocial areas | ***Intervention:***  Baseline n= 12 preschools, 84 dyads with 79 dyads at followup.  Children's mean age (SD): 3.78 (0.75) years  Boys: n=43 (51.2%)  Mother’s Mean age (SD) 34.52 (6.38) years  n= 77 (91.7%)  Father’s mean age (SD): 39.54 (8.23) years  n= 4 (4.8%).  ***Control:***  n= 12 preschools, 107 dyads with 95 dyads at follow up.  Children's mean age (SD): 3.77 (0.72) years  Boys: n= 69 (64.5%)  Mother’s Mean age (SD) 33.76 (6.94) years  n= 98 (91.6%)  Father’s mean age (SD): 40.62 (8.71) years  n= 4 (3.7%). | Parent Ambassadors (PA) visiting in pairs | ***Intervention group***  Health education – Home visits   - Structured lesson plans including   Mini lectures, flipcharts Role play to enable participants to master parenting micro skills.  Parents required to complete homework activities 5 days a week with their children in order to put parenting skills into practice  ***Control group***  Six series of parent talks in group sessions at preschool | ***Behavioural***  Oral health  questionnaire developed by  Department of Health, Hong Kong SAR Government with three questions on teeth brushing, and parents rate each item on a 5-point scale. A higher score indicates more frequent teeth brushing.  Feeding practices  Knowledge, attitude and practices assessed using Hong Kong Parent Feeding Questionnaire using 3 point Likert scale. High score indicates desirable feeding practices | ***Oral Health- tooth brushing***  Statistically significant difference found between the mean tooth brushing of intervention vs. control groups (10.55 vs 9.29, p= 0.003)  Effect size (d): 0.52 (0.18, 1.50).  ***Feeding practice***  Statistically significant difference between mean feeding practices scores for intervention vs. control groups (50.98 vs. 47, p <0.001)  Effect size (d): 0.56 (0.26, 0.85). | Low risk |  |
| **Braun et al.**  **(2016)**  **Cluster randomised controlled trial** | **USA**  To assess the  effect of an OHP program on caries, as delivered in Navajo Nation Head Start by trained Navajo community oral health specialists. | ***Intervention:***  20 HS, 528 dyads at baseline with 518 at follow up.  Children mean age (SD) 3.7 (0.03) years with 51% females.  77.5% mothers with mean age of 32.7 (SE 0.5) years  ***Control:***  19 HS, 502 dyads at baseline with 498 at follow up.  Children mean age (SD) 3.7 (0.04) years with 49.1% females.  76.5% mothers with mean age of 31.1 (SE 0.5) years | 8 trained tribal community members called Community Oral Health Specialist (COHS). | ***Intervention group***  Oral health promotion (OHP)  highly personalized set of oral health–focused interactions (5 for children and 4 for parents), along with 4 fluoride varnish applications delivered each year during academic years of 2011 to 2012 and 2012 to 2013.  Caregiver's OHP activities began with a kick-off event for caregivers and children that introduced the project. The remaining three parent events, which occurred at various times and locations to maximize attendance, included 1) an overview of the importance of primary teeth, prevention of tooth decay, consequences of tooth decay, and caregivers' roles in prevention; 2) two small-group OHP activities; 3) a simple goal-setting activity; and 4) a fruit basket raffle for enrolled caregivers who attended.  Fluoride Varnish (FV)  FV and child OHP events were delivered in the Head Start classroom  TB & TPs  All families received toothbrushes and toothpaste for all  family members at enrolment; intervention children and participating caregivers received additional supplies throughout the study period during data collection events.  ***Control group***  Received TB and TP for the whole family s at enrolment | ***Clinical (Primary outcome)***  dmfs in primary teeth at baseline+3 years follow up  ***Secondary outcomes (clinical + behavioural)***  Longitudinal assessments  of ds and decayed, missing, or filled surfaces (DMFS;  permanent dentition) counts, caries prevalence, and validated survey items assessing caregiver oral health knowledge | ***Clinical***  dmfs, ds and DMFS: No statistically significant difference for mean dmfs, ds and DMFS between intervention and control groups for all three years of follow up (p>0.05).  Effect size (d):  dmfs at Year 3: -0.04 (-0.30, 0.22)  ds at Year 3: 0.13 (-0.13, 0.39)  DMFS at Year 3: 0.0 (-0.26, 0.26)  ***Behavioural***  Statistically significant difference between mean scores of intervention vs. control group in first follow up when original caregiver completed the survey (62.7 vs. 58.2, p=0.003)  Effect size calculation (d):  Year 3: -0.04 (-0.31, 0.22)  ***Oral health Knowledge***  No statistically significant difference was reported for intervention vs. control group in mean knowledge scores for all three years.  Effect size calculation (d):  Year 3: -1.06 (-1.34, -0.78) | Low risk |  |
| **Batliner et al. (2018)**  **Randomised Controlled Trial** | **USA**  To evaluate the effectiveness of MI to reduce caries occurrence and progression among AI children < 3 y of age | ***Intervention***  n= 301 at baseline with 232 at follow up dental screening  Children mean age (SD): 0.62 (0.89) months with 47.9% females  96.9% mothers with mean age (SD): 28.2 (15.2) years  ***Control***  n= 299 at baseline with 238 at follow up dental screening.  Children mean age (SD): 0.73 (0.91) months with 54.3% females  95.8% mothers with mean age (SD): 27.5 (13.7) years  . | Local people with at least a college degree. The were called ‘MI interventionists’ and were provided with 2 days of training. | ***Intervention group:***  Received 4 MI visits and the mother selected 2 topics from a list of 8 options. For the 2 topics chosen at each visit, the mother worked with the MI interventionist to discuss her ambivalence, concerns, or hesitations and to establish goals and a plan of action. In subsequent visits, the mother and interventionist discussed progress and obstacles and then amended goals and action plans, discussing new topics as needed. At a mother’s request, the same topic could be repeated in a subsequent session, although at least 1 new topic was added in these cases.  ***Control group:***  Enhanced Community Services (ECS) included public service announcements on the tribal radio station, billboards, distribution of brochures focused on behavioural risk factors for ECC and oral health topics covered in the MI sessions. Each participant received oral health brochures according to age group and toothbrushes and toothpaste  for all family members.  ECS was provided to both intervention and control group. | ***Clinical (Primary outcomes))***  dmfs measure of decayed,  missing, or filled primary  tooth surfaces (white spot lesions were not considered) at enrollment and  when the child was 12, 24, and 36 mo of age.  ***Clinical + behavioural (Secondary outcomes)***  Longitudinal assessments  of decayed surfaces (ds) and caries prevalence at 12, 24 and 26 months.  Survey assessing  mothers’ oral health knowledge  and parental oral health behaviours at baseline and 12, 24 and 26 months. | ***Clinical***  Difference between intervention and control groups for mean dmfs (10 vs 10.4, p= 0.7), mean ds (3.2 vs 4.1, p= 0.38) was reported to be not significant.  Effect size (d) was found to be not significant  dmfs: -0.02 (-0.21, 0.16)  ds: -0.13 (-0.31, 0.05)  ***Behavioural***  Oral Health Behaviour  No significant difference reported between intervention and control for mean oral health behaviour score (53 vs 51.3, p= 0.86)  Effect size (d): 0.09 (-0.09, 0.26).  Oral Health Knowledge  Difference between intervention and control groups for mean oral health knowledge scores was reported to be significant (82.1 vs 80.1, p= 0.03), however effect size calculation showed no significant effect (d)= 0.15 (-0.03, 0.33). | Low risk |  |
| **Henshaw et al. (2018)**  **Cluster Randomised Controlled Trial** | **USA**  To assess the 2-y effect of MI on caries increment in primary teeth, as delivered by trained public housing residents to caregivers of children aged 0 to 5 y living in Public Housing Developments (PHDs). | ***Intervention***  Enrolled n= 574 with 379 dyads at follow up  Children's mean age (SE): 2.8 (0.09) years with 50.7% males.  Caregivers mean (SE) age: 32 (0.32) years with 97.9% females  ***Control***  Enrolled n= 847 with 686 dyads at follow up.  Children's mean age (SE): 2.8 (0.04) years with 51% males.  Caregivers mean (SE) age: 31.8 (0.46) years with 98.5% females | Local people living in (PHDs) recruited and trained as Oral Health Advocates (OHA)  They received 8h classroom training followed by written test and 4 wk training on delivery of MI sessions. | ***Intervention group***  Clinical examination and dental referral  On-site child clinical examinations to collect data on dmfs, with a report on current oral health status and a dental referral list; and fluoride varnish  application.  MI and other materials  Participants were presented with a menu of ECC prevention strategies to discuss with the OHA: bottle and sippy cup use; cleaning your child’s mouth; drinking fluoridated water; good-bye bottle, hello sippy cup; healthy snacks, keeping germs away; lift the lip; sleep time routine; and visiting the dentist.  Toothpaste and toothbrush along with written handouts were also provided.  ***Control group***  They received the same clinical examination and dental referral, written handouts and toothpaste & tooth brush except the MI sessions. | ***Clinical***  dmfs over time measured at baseline, 12 and 24 months  ***Behavioural***  Oral health behaviours and knowledge measured at baseline, 12 and 24 months | ***Clinical***  No significant difference reported in mean dmfs scores between intervention and control groups at 24months (3.1 vs 3.1, p= 0.53)  Effect size (d)= 0.00 (-0.14, 0.14)  ***Behavioural***  Oral Heath Behaviours  No mean difference reported between intervention vs control group in mean sugar sweetened beverage intake (2.1 vs 2.3, p= 0.43).  Effect size (d): -0.13 (-0.26, 0.02), and proportion of children with twice daily tooth brushing (OR 1.3 (0.89, 1.93, p= 0.22) at 24 months follow-up.  Oral Health Knowledge  Significant difference reported between mean scores for intervention and control groups (77.3 vs 75.9, p= 0.03) at 24 months.  ES (d): 0.07 (0.07, 0.35). | Low risk |  |
| **Villena et al.**  **(2019)**  **3- arm cluster randomised controlled trial** | **Peru**  To investigate the effectiveness of an oral health promotion, prevention, and  restoration program directed at newborns and their parents that is implemented by nurses and dentists employed at MCH clinics and fully integrated into their daily routines | ***Total sample (baseline)***  368 participants with 50.7% males and mean age (SD) of 3.1 (0.2) years.  ***Intervention***  Active Group (AG) n=128 at baseline and 70 at followup  Passive Group (PG***)*** n= 120 at baseline and 76 at followup  ***Control Group (CG)***  n= 120 at baseline and 73 at follow up | Nurses and dentists | ***Intervention***  AG: Nurses trained to advise family members during regular child visits using 3 age related oral health information cards. A time-related activity record card was also provided to assist nurses in the kinds of activities that need to be performed on a particular visit. Children with signs of caries were referred to the health centre dentists who were trained to apply FV and Atraumatic Restorative Technique (ART) sealants and restorations and emphasised importance of oral hygiene and good dietary behaviours.  PG: Nurses received the same printed materials but without any training. Dentists received trained as in the AG group.  ***Comparison***  In CG nurses only received 45 minutes lecture on importance of oral health and used the regular standard protocols. No training in ART was provided to the dentists. | ***Clinical***  Caries status using CAST index after 1, 2 and 3 years in AG and 2, 3 years in PG and CG. | ***Clinical***  Mean dmft scores calculated from CAST index were found to be significantly different for AG vs CG (Effect size g= 0.86 (0.52, 1.21) and for AG vs PG (g= 0.91 (0.57, 1.25).  No significant difference was found for PG vs CG (g= 0.07 (-0.25, 0.40) | Unclear risk |  |
| **Unkel et al. (2020)**  **Randomised Control Trial** | **USA**  To determine which mode of education given to mothers of newborns resulted in the greatest compliance for establishing an age one dental visit and identify reasons why mothers do not bring their child for their first dental visit. | ***Intervention***  1. Nurse (written) group: n= 122 at baseline and 70 at follow up  2. Nurse (written + verbal) group: n= 120 at baseline and 62 at follow up.  3. Paediatric dental resident group: n= 141 at baseline and 78 at follow up.  **Control**  **n**= 120 at baseline and 60 at follow up. | Nurses and paediatric dental resident | ***Intervention***  Group 1: A nurse provided handouts to the mothers  Group 2: A nurse provided both written handouts and discussed it with the mothers.  Group 3: A paediatric dental resident provided and discussed the same 2 informative handouts.  ***Control***  No intervention | ***Behavioural***  Intend to/Attended the dentist at child's 1 year checkup at 9-12 month followup | ***Behavioural***  Mothers in the Group 2 were more likely to have attended/intend to attend the dentist for their child as compared to the control group (OR 3.02 (1.29, 7.09).  There were no significant difference for Group 1 and Group 3 as compared to the control group (OR 2.13 (0.99, 4.54) and OR 1.78 (0.84, 3.78) respectively | Low risk |  |
| **Quasi-experimental study designs** | | | | | | | | |
| **Bentley et al. (1993)**  **Pre-Post test** | **United Kingdom**  To evaluate the effectiveness of health visitors in encouraging dental attendance of children aged 0-2 years | 7/9 districts agreed to participate  255 HVs  362 dentists agreed to accept infants.  3165 parents of children contacted during the study period | Health Visitors | ***Intervention***  Oral health advice and dental referral for registration  Every mother that HV saw was to be advised about importance of oral health and provided with simple guidelines about care of erupting teeth and importance of registering with a dentist. Any mother who was not an attendee was asked to select a dentist from the list of those accepting infant patients and then HV filled the details on a pre-printed referral letter and handed to the parent and entered the details on an evaluation form for record | ***Behavioural***  Dental registration rate | 2412 out of 3165 contacted were referred to General Dental Practice and out of these 1872 could be traced so registration rate was found to be 21% | Moderate risk |  |
| **Sgan-Cohen et al. (2001)**  **Quasi-experimental** | **Israel**  To measure the effect of a community health education program on reported infants' bottle-feeding practices and infants' toothbrushing behavior, with or without distribution of toothpaste and toothbrushes**.** | ***Total sample (baseline):*** 883 infants, 6-12 months old.  ***Intervention (n= 412 at follow up)***  Program 1 (OHE+ TP & TB): n= 169 at follow up  Program 2 (OHE only): n= 118 at follow up  ***Comparison (n= 239 at follow up)***  Control 1 (TP & TB): n= 82 at follow up  Control 2 (Without TP & TB): n= 80 at follow up. | Nurses at MCH centres | ***Intervention group***  Oral health education +TP & TB  OHE to be provided during health education on dietary, toothbrushing and dental visiting behaviours.  Toothpaste and Toothbrush for children were also provided thrice during the study period.  ***Comparison group***  HE with TP & TB distribution  No structured OHE education provided but nurses were instructed not to deny knowledge if any caregiver specifically asked about oral health. | ***Behavioural***   - Bottle use during and between meals and during sleep; toothbrushing behaviours. - Measured 6 months later through questionnaires administered during interviews of parents. | ***Behavioural***  Bottle Use   - No significant difference between intervention and control group was reported and found in effect size calculation for ‘during meals’ bottle use (OR 1.12 (0.81, 1.56), p= 0.23) and bottle use with added sugar (OR 1.69 (0.92, 3.13), p= 0.06); ‘during sleep’ bottle use (OR 1.04 (0.72, 1.52), p= 0.62) and bottle use with added sugar (OR 1.11 (0.53, 2.35), p= 0.91); ‘between meals’ bottle use (OR 1.04 (0.72, 1.52), p= 0.64). - Significant difference was reported for between meals bottle use with added sugar (p= 0.03), however effect size calculation was not significant (OR 1.11 (0.53, 2.35).   Toothbrushing behaviours  Significant differences were reported between P1 and P2 (p=0.012), P1 vs C1 (p= 0.044), P1 vs C2 (p= 0.00016).  No significant differences were reported for P2 vs C1, P2 vs C2 and C1 vs C2.  Effect size calculation:  P1 vs P2: OR 1.68 (1.05, 2.71)  P1 vs C1**:** OR 1.85 (1.09, 3.15)  P1 vs C2**:** OR 3.16 (1.80, 5.54) | Serious risk |  |
| **Harrison & Wong (2003)**  **Quasi-experimental with comparison group** | **Canada**  To design, implement and evaluate a culturally sensitive oral health promotion program to improve dental health in Vietnamese preschool children in Canada. | Baseline n= 14  Children's age (SD): 25.3 (6.2) months  ***Experimental:*** n=16  Children's age (SD): 22.1 (5) months  ***Comparison****:* Similarly aged Vietnamese children from a neighbouring municipality (n= 9).  Children's age (SD): 22.7 (5.8) months.  Baseline examinations conducted in 1994 and then comparison made of similar aged children in baseline with children in intervention groups at 1996, 1998, 1999 and 2001. However due to very small number of participants attending the oral examinations, it was excluded from analysis. | Vietnamese Lay health worker termed ‘Community Dental Health Worker’ (CDHW) | ***Intervention group***  Oral health education – Counselling+ kits+ follow-up   - CDHW provided counselling at each recommended immunization schedule (at 2, 4, 6, 12 and 18mo) and provided Oral health promotion kits provided for infant at each visit. - Follow-up phone calls to mothers for coaching and support.   Community-wide initiatives   - Dissemination of videos and articles through community-wide initiatives and other activities such as: child dental health booths at local festivals, window displays near bus stops and child oral care brochure for nurses.   ***Comparison group***  Did not receive Oral Health Education initiatives. | ***Clinical***  Presence of visual decay through defs index.  ***Behavioural***  Brief questionnaires during baseline and follow-up to determine parenting practices, awareness of community outreach activities and suggestions for improving oral health of children. | ***Clinical***   - Significant difference in mean defs between INT vs baseline at 1^st^ follow up in 1996 (1.1 (4.3) vs. 5.1 (7.2), p= <0.05).   Effect size (d): -0.69 (-1.40, 0.07) not significant.   - Significant difference between INT vs baseline at 2^nd^ follow up in 1998 (0.06 (0.2) vs. 9.5 (10.9), p= <0.05).   Effect size (d): -1.18 (-1.85, -0.45).   - Significant difference between INT vs baseline at 3rd follow up in 2001 (2.6 (2.8) vs 9.5 (10.9), p <0.05).   Effect size (d): -0.83 (-1.49, -0.12) significant  ***Behavioural***   - Significantly less use of day time and sleep-time bottle use for children 12-60 mon of age, reported by mothers with > 1 counselling sessions, (*p*<0.005).   Effect size:  Daytime bottle use (OR): 0.03 (0.005, 0.18)  Sleep time bottle use (OR): 0.07 (0.01, 0.36) | Moderate risk |  |
| **Yuan et al. (2007)**  **Quasi-experimental non-equivalent two group comparison** | **Ireland**  To evaluate the effectiveness of a community-based program to promote dental registration and access to dental services for preschool children residing in areas of high social deprivation using monthly registration data provided by the Central Services Agency (CSA). | ***Intervention:***  9 wards out of which 3 were urban and 6 rural.  ***Comparison:***  14 wards out of which 6 were urban and 8 rural | Community-based nurses (health visitors) (n=12) and General  Dental Practitioner (GDPs) (n=44) | ***Intervention group***  Oral health education+ Dental referral: Home visits  Health visitors providing dental health education + oral health promotion kit containing feeding cups, toothbrushes and fluoride toothpaste+ registration vouchers and list of dental practices taking part in the initiative.  GDP appointment  GDP provided advice on how to care for baby’s teeth, regular dental attendance and maintenance of child’s dental registration.  Vouchers provided to mothers were exchanged for motivational materials such as wipe-clean table mat and a height chart).  ***Control group***  Received usual care. | ***Behavioural***  Dental registration rates for preschool children (0-2y and 3-5y)  Measured rates at 3 time points:  baseline (6months before program), T1 (during the program), and T2 (5months after program completion). | ***Behavioural***  T1: The mean registration rate of children 0-2yr in the intervention wards was not significantly different from controls  Mean difference 0.03 (-0.02, 0.09) p 0.21).  During the programme, the rate of change of registration (slope) for 0-2 year old children was significantly greater compared to rate of change in control wards  Mean difference: 0.005 (0.002,0.007)   - T2: Statistically significant difference between mean registration (mean difference 0.04 (-0.08, 0.00): P <0.05). - There were no statistically significant effects for the 3-5yr group. | Low risk |  |
| **Kressin et al. (2009)**  **Quasi-experimental design** | **USA**  To assess the effects of training paediatricians in providing patient centered counselling intervention on provider ECC counselling  practices, and on children’s subsequent development of ECC. | ***Intervention:***  Baseline n = 635 with n= 607 parent child pairs at follow up.  ***Comparison:***  Baseline n = 452 with n= 438 at follow up.  Children:  Intervention: 1.93 y, 51% males  Control 1.87 y, 53% males | 13 Paediatricians  14 Clinic Nurses (RNs and NP) | ***Intervention group:***  3 components:  (1) Communication skills training to enhance clinicians’ ability to counsel participants and they were trained to address 3 primary dimensions with caregivers: advice about diet, hygiene and tooth monitoring to detect development of caries. Providers were asked to implement 4A's: Assess parents' status on each dimension, Assist with addressing the barriers, Advise about ECC and Arrange follow up (2) Edits to EMR to include age appropriate information for each dimension and (3) educational brochure to be handed out by clinicians during consultation with caregivers.  ***Comparison***  Brochures were made available. | ***Clinical***  Incidence of ECC at 1 year follow up | ***Clinical***  No statistically significant difference between proportion of children with ECC in intervention and comparison site (17.7% vs. 31.7%) p= 0.086.  Survival analysis using Hazard Ratio shows significant results: 0.23 (0.09, 0.62), p= 0.004. | Moderate risk |  |
| **Nair et al.**  **(2009)**  **Pre-post test** | **India**  To evaluate the effectiveness of a community oral health awareness  program given to mothers through trained community level workers (Junior Public  Health Nurses (JPHNs) and Anganwadi workers (AWWs). | 232 mothers of children aged 0-6 years. | Junior Public Health Nurses (JPHNs) and Aganwadi Workers (AWWs) n= 115 | ***Intervention***  Training materials for health workers included: audiovisual aids such as modules, charts, posters and brochures in regional language. Modules and booklets were used to help impart health sessions  to mothers during sessions at Aganwadis. | ***Behavioural***  Mother’s knowledge regarding children’s oral health | ***Behaviour***  Questions on knowledge about oral hygiene such as when to start toothbrushing in children (p= 0.002), knowledge about importance of milk teeth: conservation of milk teeth is essential (p <0.001), reason for conserving milk teeth (p= 0.006) and knowledge about causes of dental disease: Causative factor in caries (p <0.001) showed significant improvement in scores, all showed significant improvement  Effect size (RRR 0.28 (0.13, 0.40), 0.69 (0.54, 0.79), 0.21 (0.07, 0.33), 0.27 (0.19, 0.33) respectively | Serious risk |  |
| **Maupomé et al (2010)**  **Quasi experimental** | **USA**  To outline caries changes after 18-to-30 months of follow-up in  children from AI communities who were exposed to family-based and/or community-wide  interventions, or served as a regional comparison group. | Post-intervention sample  ***Interventio***n  Community A:  Female: n= 23, mean age (SD) 23 (3.6) months  Male: n= 23, mean age (SD) 24.3 (3) months.  Community B  Female: n= 17, mean age (SD) 26.6 (2.9) months  Male: n= 20, mean age (SD) 22.9 (2.8) months.  Community C  Female: n= 23, mean age (SD) 23.1 (2.8) months  Male: n= 27, mean age (SD) 23.3 (2.7) months.  ***Comparison***  Community D  Female: n= 17, mean age (SD) 24.7 (3.3) months  Male: n= 25, mean age (SD) 25.1 (3.1) months. | Community health workers (CHWs) provided the family interventions based on home visiting model | ***Intervention***  Community based and family interventions  Media based community-wide interventions were designed in six-month cycles, using five strategies: (i) raising awareness, (ii) providing health education, (iii) facilitating individual behaviour change, (iv) augmenting public health practice, and (v) modifying environments or policies related to breastfeeding, sugar-sweetened beverages, and water consumption. A sample community-wide intervention plan targeting sugar-sweetened beverages.  Family Interventions were delivered in eight visit clusters by community health workers (CHWs) using a home-visiting model. CHWs created a client-specific plan for initiating and maintaining breastfeeding along with water and sugar-sweetened beverage interventions in clusters 1–3. Cluster 1 occurred before the baby’s birth, to facilitate counseling that would encourage  early decisions to breastfeed. Clusters 2–4 occurred within 0–3 months of the baby’s birth. Clusters 4–7 consisted of intervention implementation and final data collection was done in cluster 8.  ***Comparison***  No intervention | d1 and d2 components of dmft index as no teeth were missing or filled. | All three intervention communities showed substantial improvements in d1t and d2t.  Effect size (d):  d1t:  A vs D: -0.52 (-0.94, -0.09)  B vs D: -0.64 (-1.09, -0.18)  C vs D: -0.35 (-0.76, 0.06)  d2t:  A vs D: -0.42 (-0.84, 0.01)  B vs D: -1.17 (-1.64, -0.68)  C vs D: -0.18 (-0.59, 0.23) | Moderate |  |
| **Vichayanrat et al.**  **(2012)**  **Quasi exp pre-post-test with comparison group** | **Thailand**  To dem­onstrate the application of the SEM to oral health interventions and to evaluate its ef­fects on oral health practices among care­givers and their determinants at multiple levels | ***Intervention:*** 62 parent child pairs  Children's mean age (SD): 19.16 (8.74) months.  Caregiver's mean age (SD): 31.74 (10.61) years.  ***Comparison:*** 52 pairs  Children's mean age (SD): 18 (9.30) months.  Caregiver's mean age: 34.50 (11.52) years. | Health Centre staff and Lay Health Workers (LHWs) | ***Intervention***  1. Oral health education & services at health centres:  Health centre staff provided 4 main activities: oral health screening, structured oral health education, prescribing fluoride supplements and giving toothbrush and/or toothpaste.  2. Home visits by LHWs to provide social support:  Trained LHWs visited every 3 months to provide social support: informational, appraisal and emotional support for caregivers.  3. Community mobilisation process:  members of Tambon Administrative Organisation and Day care teachers and village health volunteers were invited to meetings during the programme to educate them and group discussions to better understand ECC problem and its prevention  ***Comparison***  Received routine health services from local health centres, and toothbrushes pre- and post-test. | ***Clinical***  Caries status in children assessed by dmft.  ***Behavioural***  caregiver's oral health knowledge and behaviour | ***Clinical***  No significant improvement in mean dmft was reported for intervention and comparison group (3.04 vs 3.49, p=0.993).  Effect size (d): -0.11 (-0.48, 0.26).  ***Behavioural***  Tooth brushing***:***  Significant improvements were seen for ‘any toothbrushing during the previous week’ between INT and comp group (93.5% vs. 80.4%, p= 0.035)  Effect size (OR)= 3.45 (1.01, 11.76)  Use of fluoride tooth paste. Brushing with adult supervision was not found to be significant.  Bottle Feeding:  No significant improvements seen for behaviours of ‘falling asleep with bottle’ and ‘putting sweetened milk, juice or soda in the bottle’. ‘Use of fluoride supplements’ showed significant improvement 80.3% vs. 13.7%, p <0.0001)  ES (OR) 24.23 (8.88, 66.13).  Cariogenic snack consumption (>4 days)  There was a significant decrease in low cariogenic snack consumption only for INT vs. comp group 46.8% vs. 65.4%, p= 0.058)  Effect size: (OR) 0.46 (0.22, 0.99).  ***Knowledge***  Significant improvement for only two knowledge items such as ‘method of using fluoride supplements’ (57.6% vs. 13.5%, p <0.0001) and ‘not putting juice in bottle’ (66.1% vs 34.6%, p 0.001) was reported.  Effect size (OR) 8.9 (3.47, 22.85) and 3.69 (1.70, 8.02) respectively. | Serious risk |  |
| **Raj et al.**  **(2013)**  **Pre-post test** | **India**  To evaluate the short-term impact of Oral Hygiene Training Package (OHTP) to AWWs on improving oral hygiene of preschool children. | ***Pretest (n= 534 children)***  Females: n= 263 (49.3%)  Males: n= 271 (50.7%)  ***Posttest (n= 538 children)***  Females: n= 246 (45.7%)  Males: n= 292 (54.3%) | Aganwadi Workers (AWWs)  n= 21 | ***Intervention***  AWWs imparted oral health education to mothers in weekly meetings in their respective AWCs for 12 weeks. Meetings were conducted guided by the training module , posters and stories given to AWW as part of OHTP | ***Clinical***  Changes in plaque index scores, gingival index scores, debris index and caries activity.  ***Behavioural***  Improvements  in oral hygiene practices (frequency of tooth  brushing, mouth rinsing after meals) | ***Clinical***  No significant differences reported between pretest and posttest for mean dmft scores (2.1 vs.1.9, p= 0.06).  Effect size (d): -0.08 (-0.20, 0.04).  Significant reduction reported in caries activity (n) between pre and post-test assessed through Snyder’s test (241 vs. 168, p<0.05).  Effect size: RRR 0.35(0.24, 0.45).  Significant improvements in debris index (as objective measure of toothbrushing) also reported (n) (418 vs. 291, p <0.001), Effect size: RRR 0.31 (0.24, 0.37).  ***Behavioural***  1. Significant improvements in pre-test vs post-test for brushing twice or more (%) (4.1 vs. 9.9, p <0.001).  Effect size: RRR 0.06 (0.03, 0.09);  and those that never brushed (13.9 vs. 7.2, p <0.001).  Effect size: RRR 0.48 (0.24, 0.64)  2. Significant improvements also seen in use of medium of cleaning between pre and post-test:  Use of tooth brush (85.2 vs. 90.2, p 0.017).  Effect size: RRR 0.33 (0.08, 0.52);  use of nothing to clean (13.9 vs. 7.2, p <0.001).  Effect size 0.48 (0.24, 0.64). | Serious risk |  |
| **Wilson et al. (2013)**  **Quasi exp pre-post test** | **USA**  The research hypothesized that family-centered, peer to-  peer videos would be more effective than didactic, lecture based  videos in achieving positive changes in family knowledge,  attitudes and behaviours related to young children’s oral  health among families with young children living in rural  Hawai’i. | Total 91  BFS video group 48  BOH video group 43  ***Children***  52% boys  48% girls | Health Visitors (n= 19) with at least 6 months experience and atleast 6 attached families. | ***Intervention***  Each video was divided into eight segments lasting four to seven minutes in duration. The segments were to be shown on an overlapping schedule. Home visitors were asked to complete the series within eight to ten weeks. During the video intervention period, home visitors continued to implement the ongoing EHS protocol. How and when to incorporate the video during each home visit was left to the home visitor’s discretion. To minimize differences in presentation, home visitors were asked to simply play the video and to provide only brief answers to any questions from family members. Home visitors were asked to refrain from showing the non-assigned video or from designing and implementing any supplementary curriculum. When each family had completed the video series, the home visitor was asked to give the participant a copy of the assigned video and encourage the family to share the video with other parents in their social circle. | ***Behavioural***  Family oral health knowledge, attitudes and behaviours related to young children's oral health | ***Oral health Behaviours/attitudes***  When evaluated individually, the BOH or BFS did not have any statistically significant effect in changing behaviour/attitudes between pre and post-test.  (Effect size (d): 0.27 (-0.16, 0.69) and 0.36 (-0.04, 0.76) respectively).  When mean scores were evaluated between pre and post test for both videos, there was a significant improvement reported in oral health behaviours/attitudes (Mean scores pre vs. post for total: 103.70 vs. 107.02).  Effect size (d): 0.31 (0.02, 0.61), p= 0.0005).  ***Oral health Knowledge***  Significant improvement in mean knowledge scores were seen between pre and post scores for BOH and BFS individually and also for the effect when both scores were averaged.  Mean scores: BOH pre vs. post: 15.30 vs. 17. 01; BFS pre vs. post 15.08 vs. 16.95 and for total: 15.19 vs. 16.98.  Effect Size (d):  BOH pre and post: 0.52 (0.09, 0.95),  BFS pre and post: 0.52 (0.11, 0.92)  Total: 0.52 (0.23, 0.82) p= 0.0005 | Serious risk |  |
| **Van den Branden et al.**  **(2013)**  **Quasi experimental with control group** | **Belgium**  To evaluate the effectiveness of a multi-component oral health intervention in preschool children in a non-randomized intervention study with a complementary baseline control | ***Intervention***  Baseline n= 1284 and n= 1080 at follow up.  at 3 year's age follow up:  mean age 3.1 year  at 5 year's age follow up:  mean age 5.20 years  52.4% boys.  Mother's mean age (at child's birth): 28.7 years.  ***Control***  Baseline n = 1171 and n= 1057 at follow up.  at 3 year's age follow up:  mean age 3.2 year.  at 5 year's age follow up:  mean age 5.2 year.  50.9% boys  Mother's mean age (at child's birth): 29.9 years. | 3 home visits by a nurse and 11 consultations by a physician and a nurse up to child's 3 years of age. Oral health education provided by a specifically trained nurse. | ***Intervention***  Home visits+ oral health kits  3 Home Visits: at 2, 4-6 and 10 weeks  11 consultations: from 3-6 weeks until the child is 30 months old Oral health promotion was developed and added to the standard programme by adding oral health education on 14 topics like breast-feeding, pacifier use, parental oral hygiene, water consumption, and brushing behaviour. Child health booklet, toothbrush and fluoride containing TP sample, cup and placemat were also given to parents. All health care professionals practicing in the intervention region were informed about the project and posters were provided to inform their patients.  ***Control***  Standard programme | ***Clinical***  Decay experience assessed through dmft and dmfs  ***Behavioural***  Dietary and oral hygiene behaviours,  dental attendance  Assessed at 3 and 5 years of age of child | ***Clinical***  No significant differences reported for proportion of children caries free in intervention vs control group assessed by d3mfs at 3 years follow up (97.5 vs 95.7, OR 1.25 (0.63–2.49)  Significant difference between intervention vs control for d1mfs at 3yr follow up (91.9 vs 77.9, OR 2.47 (1.70-3.59), p <0.001).  No significant difference between intervention vs control for d3mfs at 5yr follow up (76.5 vs 76.1, OR 0.99 (0.77–1.26) p= 0.92).  No significant difference between intervention vs control for d1mfs at 5year follow up (61.3 vs 60.6, OR 0.99 (0.80–1.22) p = 0.91).  ***Behavioural***  Significant differences in proportion of children who visited the dentist less than a year ago, brushed more or more with fluoride tooth paste and were assisted with brushing once or more. However better behaviours were seen for the control region both at 3 and 5 years' follow up.  Significant difference favouring intervention was reported for less than daily consumption of sugared drinks in between meals both at 3 y (53.2 vs.38.9, p <0.001) and 5 years (54.4 vs. 42.4, p<0.001).  Effect size OR at 3 years: 0.53 (0.43, 0.67), RR 0.78 (0.71, 0.85),  OR at 5 years: OR 1.62 (1.33, 1.98), RR 1.28 (1.15, 1.42)  No significant difference was reported between intervention vs control group for less than daily consumption of sugared snacks in between meals both at 3 and 5 year follow up.  When only comparing the intervention and control group, no effects of the intervention were observed, as the control group generally performed better. However, when comparing the data with a historical cohort, some small effects of the intervention program could be observed in the short term. Thus, the intervention seems to have had limited success in improving oral health-related behaviours such as visiting the dentist”, tooth brushing more than once with fluoride tooth paste, assisting with tooth brushing more than once, consumption of sugared drinks and snacks in between meals at 3 years and except consumption of sugared snacks for 5 years follow up. | Moderate  risk |  |
| **Biordi et al.**  **(2015)**  **Quasi exp pre-post test** | **USA**  To provide oral health care services at 2 sites using a nurse  practitioner–dietitian team to increase dental workforce capacity and improve access to care for low-income preschool children. | ***Visit 1*** Total n= 4360  Rural: 2493, Urban: 1867  ***Visit 2:*** Total n =1832  R = 1127, U= 705  ***Visit 3***: Total n=728  R= 492, U= 236  Age  **Visit 1:** 2.31 y**ears**  **Visit 2:** 2.79 years  **Visit 3**: 3.15 years  Gender  **Visit 1**  Female= 49%, Male= 51%  **Visit 2**  Female= 48%, Male= 52%  **Visit 3**  Female= 47%, Male= 53% | 1 Dietician and 1 Nurse Practitioner | ***Intervention***  Fluoride varnish application, oral health education discussion with parents and provision of written information and oral health assessment. Parents or guardians were also given a list of paediatric dentists who accepted public insurance, and the children received a bag containing a toothbrush, toothpaste, and age-appropriate oral health education  materials, including a dental colouring book and crayons at 3 or 6 months visit depending on WIC procedure for 3 visits between the study period of 2010-2013. | ***Clinical***  Mean decayed, discoloured or filled teeth  ***Behavioural***  Oral hygiene and dietary habits  Dental visits  Assessed at 3 visits between 2010-2013 | ***Clinical***  No significant differences reported for mean decayed, discoloured or filled teeth between visit 1, 2 and 3  ***Behaviour***  Significant differences reported between rural and urban sites for child drinks > 1 cup of sweet drink/day; child uses bottle for milk and other drinks; child uses sippy cup for milk and drinks; child eats fruits/veggie at least 1time/day and child snacks on high sugar foods more than 1 time/day for all 3 visit except for child uses sippy cup for visit 3.  Effect size: calculation not possible with the provided information. | Serious risk |  |
| **Gibbs et al.**  **(2015)**  **Community Trial** | **Australia**  The aim of the exploratory trial was to establish a model for feasible, replicable and affordable child oral health promotion for culturally diverse Local Government Areas (LGAs) in Australia | ***Intervention***  Baseline n= 288 families/ 378 children and n=154 families with 197 children at follow up.  Caregivers:  mean age: 33.2 years Females: n= 164 (83%) Males n= 33 (17%).  Children:  Females: n= 100 (51%) Males n= 97 (49%).  ***Control***  Baseline n= 233 families/314 children and n=110 families with 144 children at follow up.  Caregivers:  mean age: 33.5 years Females n= 112 (78%)  Males n= 32 (22%)  Children:  Females: n= 76 (53%) Males n= 68 (47%). | Peer educators who were members of the community and fluency in both English and their ethnic language. | ***Intervention- ‘Teeth Tales’***  OHE + OH pack + reminders  Two 3 h sessions of oral health education followed by a site visit to the local community health dental service to be familiarised with the service and other local family services.  Provision of OH pack containing oral health information, toothbrush and toothpaste for the whole family.  Reminders (1 message per month for 4 months) either by text or post according to preference.  ***Comparison***  Families recruited from outside the study area were treated as the comparison group | ***Clinical***  Assessment of debris and gingival index as proxy measures of oral hygiene.  Decay assessment using dmfs index.  ***Behavioural***  Self-reported measures of child's tooth cleaning frequency and oral health knowledge and use of dental services | ***Clinical***  No significant difference reported for mean dmfs scores between INT and comp. groups (p >0.05).  Effect size (d): 0.15 (-0.08, 0.38).  Significant differences between INT and comp group were reported for presence of tooth debris (OR 0.44 (0.22, 0.88), p= 0.021), and presence of gingival inflammation (OR 0.34 (0.19, 0.61), p <0.001).  ***Behavioural***  Toothbrushing behaviours  No significant difference was reported for twice daily toothbrushing behaviours between INT and comp groups (OR 1.41 (0.77, 2.58), p= 0.259).  Dietary behaviours  No significant difference for  frequency of consumption of  cariogenic drinks several times/day (OR 1.17 (0.76, 1.80)), Freq of cariogenic foods served/day (OR 0.65 (0.40, 1.05)),  Addition of sugar to child’s drink sometimes/always (OR 1.00 0.62, 1.62)), addition of sugar to child's food sometimes/always (OR 0.97 (0.61, 1.56))  Dental visiting  No significant difference for child's dental visit (OR 0.96 (0.64, 1.45).  Oral Health Knowledge  Oral health knowledge did not show any significant effect of the intervention (OR 0.53 (0.26, 1.05)). | Serious risk |  |
| **Hoeft et al. (2016)**  **Pre-post test** | **USA**    To determine the effectiveness of the Contra Caries Oral Health Education Program (CCOHEP) for improving low-income, Spanish speaking parents’ oral health knowledge and behaviours for their young children. | Parent/caregiver and their child closest to 3 years of age, n= 105 with n= 79 at follow up.  Caregivers  mean age (SD:) 33.7 (8) years with n=81 (77%) of them mothers.  Children  Mean age (SD:) 3 (1.3) years with n=47 (45%) being females. | Lay People with parenting/childcare experience hired and trained as *promotoras* or community health outreach workers | ***Intervention- Contra Caries Oral Health Education Program (CCOHEP)***  OHE+ TP and TB provision  Four 2 h sessions on oral health education delivered in interactive manner. Sessions included information about importance of primary teeth, good dietary behaviours and oral health behaviours and establishment of early dental visiting routine.  Toothpaste and tooth brushes were also provided for the whole family. | ***Behavioural***  Self-reported oral health behaviours such as dietary and toothbrushing behaviours and oral health knowledge.  Verbal questionnaires were administered by bilingual researchers at baseline, 1 month later (post-test 1) and then 3 months later (post-test 2). | ***Behavioural***  Toothbrushing behaviours  Significant improvement was reported between baseline and post-test (p= 0.0001) for average number of correct toothbrushing behaviours, and also between post-test 1 and post-test 2 were reported (p= 0.0004).  Effect size (d): 1.55 (1.20, 1.88)  Dietary behaviours  Significant improvement was reported between baseline and post-test 1 for dietary behaviours such as child’s consumption of sweet drinks once a day or less (p= 0.0082), however this decreased non-significantly between post-test1 (77%) and post-test 2 (63%), p= 0.1306.  Effect size (RRR): 0.45 (0.23, 0.60)  However, no significant improvements in child’s consumption of sweet foods less than everyday was reported from baseline and post-test1 (0.2568) and also between post-test 1 and post-test 2 (p= 0.8575).  Effect size (RRR): 0.14 (-0.13, 0.34).  Oral Health Knowledge  Significant improvement was reported for mean knowledge score between baseline (12.8) and post-test 1 (15.2), p= <0.0001 and insignificant reduction between post-test 1 and post-test 2 (15.2), p= 0.1797.  Effect size (d): 1.89 (1.52, 2.24). | Moderate risk |  |
| **Smith et al. (2018)**  **Quasi experimental with historic control** | **Australia**  To assess the effectiveness of dental health education program ‘Smile not Tears’ in preventing early childhood caries in young Aboriginal children | ***Intervention***  Baseline n= 147 and n= 107 at follow up.  mean age (SD): 2.6 (5.7) years  girls n= 53 (49.1%)  ***Control***  n = 82  mean age: 2.8 years  girls n= 36 (43.9%), boys n= 46 (56.1%). | Aboriginal Health Workers (AHW) from 8 Aboriginal Community Controlled Health Services | ***Intervention***  The AHW met with parents over 5 visits  to deliver age appropriate messages at 6, 9,12, 18 and 24 months of age. Sessions included didactic and interactive delivery style.  Magnets and leaflets distributed  in 1st visit at 6 months of age, Fluoride toothpaste and toothbrush given at every  subsequent visit including 6th  visit which also included dental  examination. | ***Clinical***  The caries prevalence at 30 months of age as compared to similar aged children in the control group***.*** | ***Clinical***  The mean dmft and dmfs scores greatly differed between the intervention vs. control group (0.1 vs. 2.1 and 0.5 vs. 2.7).  Effect size (d): -0.75 (-1.04, -0.45) and  -0.63 (-0.92, -0.33) respectively | Moderate risk |  |
| **Yuan et al. (2019)**  **Quasi experimental** | **Northern Ireland**  To evaluate a culturally appropriate community-based home visiting oral health education intervention for Chinese, undocumented migrant mothers to promote their infants’ oral health, by focusing on their oral health related knowledge, attitudes, and behaviours. | ***Intervention***  Recruited 18 mother child pairs with 17 mother-child pairs at follow up.  Children:  Females n= 8 (44%)  Males n=10 (56%)  ***Control***  Recruited 18 mother child pairs with 17 mother- child pairs at follow up.  Children:  Females n= 7 (39%)  Males n=11 (61%) | 1 Chinese Health Visitor | ***Intervention***  Advice about breast feeding and weaning and children's and mother’s oral health advice at 8 weeks followed up by phone call at 4 months and then visit again at 6 months with oral hygiene and diet advice given to mothers then follow up call at 9 months. Breastfeeding and weaning leaflets+ teething ring+ mother's TP and TB provided at 4 weeks and then Baby trainer cup; Oral health pack containing baby toothbrush and fluoride toothpaste; Mother’s toothbrush and fluoride toothpaste at 6 months and baby feeding cup and mother's and child's TP and TB at 1 year. | ***Behavioural***  Mothers’ oral health related knowledge, attitudes and behaviours with regard to baby toothbrushing, sugar consumption and baby tooth decay as well as maternal dental health behaviours, measured at eight weeks, six months, and 12 months | ***Behavioural***  Toothbrushing behaviours  Significant differences between mean scores of intervention vs control group were reported for importance and intention to brush child’s teeth at 6 months 22.61 vs 18.83, p <0.001) and 12 months (22.82 vs 18.76, p <0.001).  Effect size (d): 1.71 (0.92, 2.43) and 1.62, (0.84, 2.33)  Dietary behaviours  Significant differences between mean intervention vs control group scores were reported for importance and intention to control sugar snacking at 6 months (31.11 vs. 27.22, p <0.001) and 12 months (32.59 vs 27.82, p <0.001).  Effect size (d): 1.35 (0.60, 2.03) and 1.36 (0.61, 2.06). | Serious risk |  |
| **Heilbrunn- Lang et al.**  **(2019)**  **Pre-post test** | **Australia**  To (i) assess the impacts of Tooth-Packs distribution on child and family OH behaviours and knowledge, including MCHN child referral practices  to dental services, and (ii) determine the feasibility  and acceptability of incorporating Tooth-Packs distribution  into MCHN OHP practices. | ***Intervention***  n= 698 completed the baseline questionnaire and 230 completed the follow up.  Mean age (SD) of children: 1.82 (0.36) at baseline  and 3.03 (0.92) at followup of which n= 116 (50.4%) were females and n= 114 (49.57%) males. | Maternal Child Health (MCH) Nurse | Families accessing the MCH centres were offered Tooth packs at 18- month and 24- month age visit. This contained age appropriate toothbrush and toothpaste for the whole family and information about OH and dental services.  MCHN conducted lift-the lip checks to identify oral disease child referral to oral health professional | Outcomes assessed at baseline and follow up (at child's 30 months' age)  ***Behavioural***  Oral Hygiene & dietary  Caregiver's self-reported oral hygiene behaviours, child's oral hygiene and dietary behaviours.  Dental visiting  Child having ever visited the dentist  Knowledge  Caregiver's knowledge and perceptions about oral hygiene | ***Behavioural***  Oral Hygiene  Significant difference between pre and post-test for tooth brushing behaviour of child such as caregiver assisting with brushing and brushing child's teeth using child toothpaste >once a day (OR 1.76 (1.05-3) and 2.82 (1.59-5.24) respectively).  Dietary  Significant differences were reported between pre and post intervention for juice consumption ≤once a week (OR 0.43 (0.22-0.84)). Difference in consumption of other beverages was not found to be significant (water, plain milk, flavoured milk, soft drinks).  Vegetables consumption ≥once a week was found to be significant (OR 0.38 (0.18-0.77)) whereas fruit consumption was not (OR 0.61 (0.26–1.37)).  Consumption of sweet spreads and chocolate lollies also reported to show a significant difference between pre and post-test (OR 2.63 (1.52-4.73), 0.21 (0.10-0.40) respectively).  Dental visiting  Reported to be significantly different between pre and post-test (OR 28 (7.40-236.88)).  Knowledge  No significant effect of the intervention was reported for knowledge questions such as age of initiation of child's toothbrushing (OR 1.25 (0.73–2.13)), white spots maybe a sign of tooth decay (OR 1.27 (0.76–2.12)), only water should be given in bottle when child is in bed (OR 1.6 (0.94–2.75)). Questions such as only bottle fed children get tooth decay and whether anyone has shown parents how to brush their child's teeth showed significant difference between pre and post-test (OR 2.69 (1.39–5.54)) and OR 3.67 (2.04–6.99) respectively). | Serious risk |  |
| **Villalta et al. (2019)**  **Pre-post test** | **USA**  To train Latina Community Oral  Health Workers (COHWs) using a promotora CHW model to educate underserved and minority populations in Los Angeles  County on best practices in oral health care, with the ultimate  goal of reducing ECC**.** | ***Intervention***  n= 157 caregivers  Female n= 130 (88%)  Male n= 18 (12%) | 13 COHWs | ***Intervention***  Trained COHWs (in teams of two) conducted 15 1 h  bilingual oral health promotion workshops for a convenience sample of 157 caregivers of young children in  the local community. They also included provision of free oral hygiene supplies,  small raffle gifts. Workshops were conducted at local elementary schools, parks, homes, and WIC sites. | ***Knowledge***  Change in parental knowledge and beliefs regarding children's oral health assessed immediately after the workshop session. | There was significant improvement in parental knowledge regarding:  How can tooth decay be prevented (Effect size: RRR 0.23 (0.07, 0.36), initiation of toothbrushing in children with fluoride toothpaste ( RRR 0.61 (0.47, 0.72), child's first dental visit age (RRR RRR 0.6 (0.28, 0.77) and age of initiation of toothbrushing by child themselves (RRR 0.26 (0.13, 0.37), | Serious risk |  |
| **Turton et al. (2020)**  **Quasi-experimental with comparison group** | **Cambodia**  To critically review  the feasibility of the Cambodia Smile intervention by considering  clinical outcomes, acceptability and stakeholder perceptions. | ***Intervention***  n= 262 mother-child dyads included and 186 remained in the study at 2 years followup.  Children:  Female n= 199 (53.8%)  Caregivers:  Female n= 102 (54.8%)  Male n=83 (44.6%)  ***Comparison***  n= 184 mother-child dyads  Caregivers:  Female n=97 (52.7%)  Male n= 87 (47.3%) | Primary healthcare providers (Nurses and midwives) | ***Intervention***  Provision of Oral Heath Education (OHE) through use of a pictorial flip chart. It included dietary advice (especially dietary sugar reduction) and oral hygiene instructions. Toothbrushes and fluoridated toothpaste were provided for both mother and child at the baseline and for child at subsequently. Fluoride varnish was also applied. | Followup at 2 years post intervention  ***Clinical***  Caries prevalence and plaque scores  ***Behavioural***  Dietary behaviour  ***Impacts***  Oral Health Related Quality of Life (OHRQoL) | ***Clinical***  Significant difference between intervention and comparison group for caries prevalence and plaque score: ECC-1 (white spot on enamel)  Effect size (d)= 1.43 (1.20-1.65).  ECC-3 (visible cavity extending into dentine) d= 0.30 (0.09-0.50).  Plaque score: d=1.58 (1.34-1.81)  After controlling for household income, the odds of developing white spot or any cavity when only provided with OHE (0.13 (0.40), p <0.001 and 0.54 (0.28) p= 0.034) and also when OHE + FV was provided 0.14 (0.39), p<0.001 and 0.39 (0.28), p= 0.001    ***Behavioural***  There was no significant difference in mean consumption of sugar sweetened beverages/day  Effect size, d=0 and packaged snacks/day d= 0.09 (-0.11, 0.29)  ***Impacts***  Both OHE and OHE+FV provision had intervention children significantly less likely to develop any impacts on OHRQoL when controlled for household income  OR (SD): 0.16 (0.34), p <0.001 and 0.17 (0.32), p <0.001) respectively. | Moderate risk |  |
